# Supplementary material for: Meta-analysis shows no consistent evidence for senescence in ejaculate traits across animals
Source: Nat Commun. 2024 Jan 16;15:558. doi: 10.1038/s41467-024-44768-4 (PMC10791739; doi:10.1038/s41467-024-44768-4)
Supplement: Supplementary file 1 — Supplementary Information [file 41467_2024_44768_MOESM1_ESM.pdf]

Meta-analysis shows no consistent evidence for senescence in ejaculate traits across animals

Supplementary figures

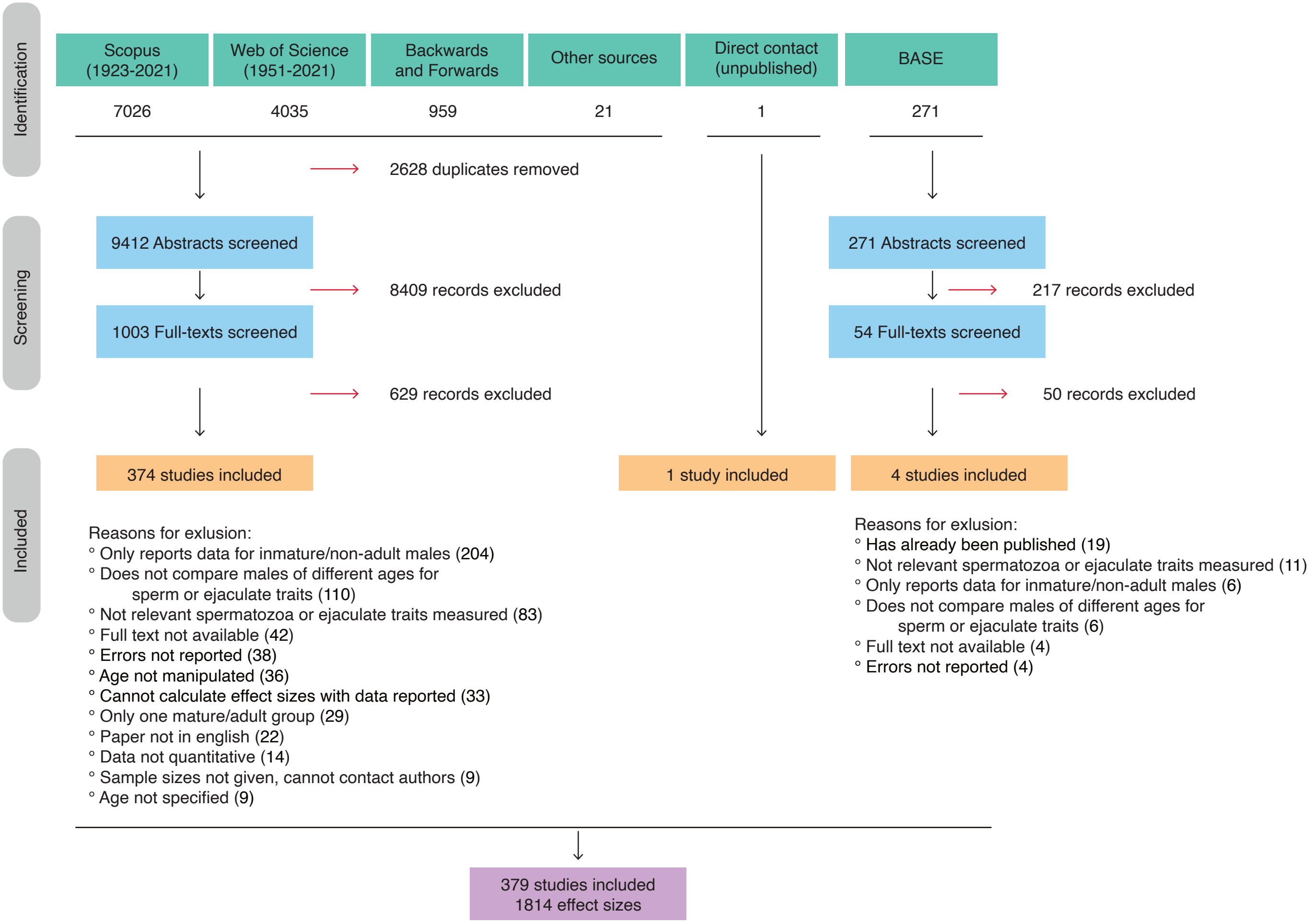

Supplementary Fig.1: PRISMA diagram describing the search results in different search engines and the different steps of selecting articles for inclusion in the meta-analysis. Depicted are the number of studies excluded at each stage and then those extracted, screened, and included in the meta-analysis.

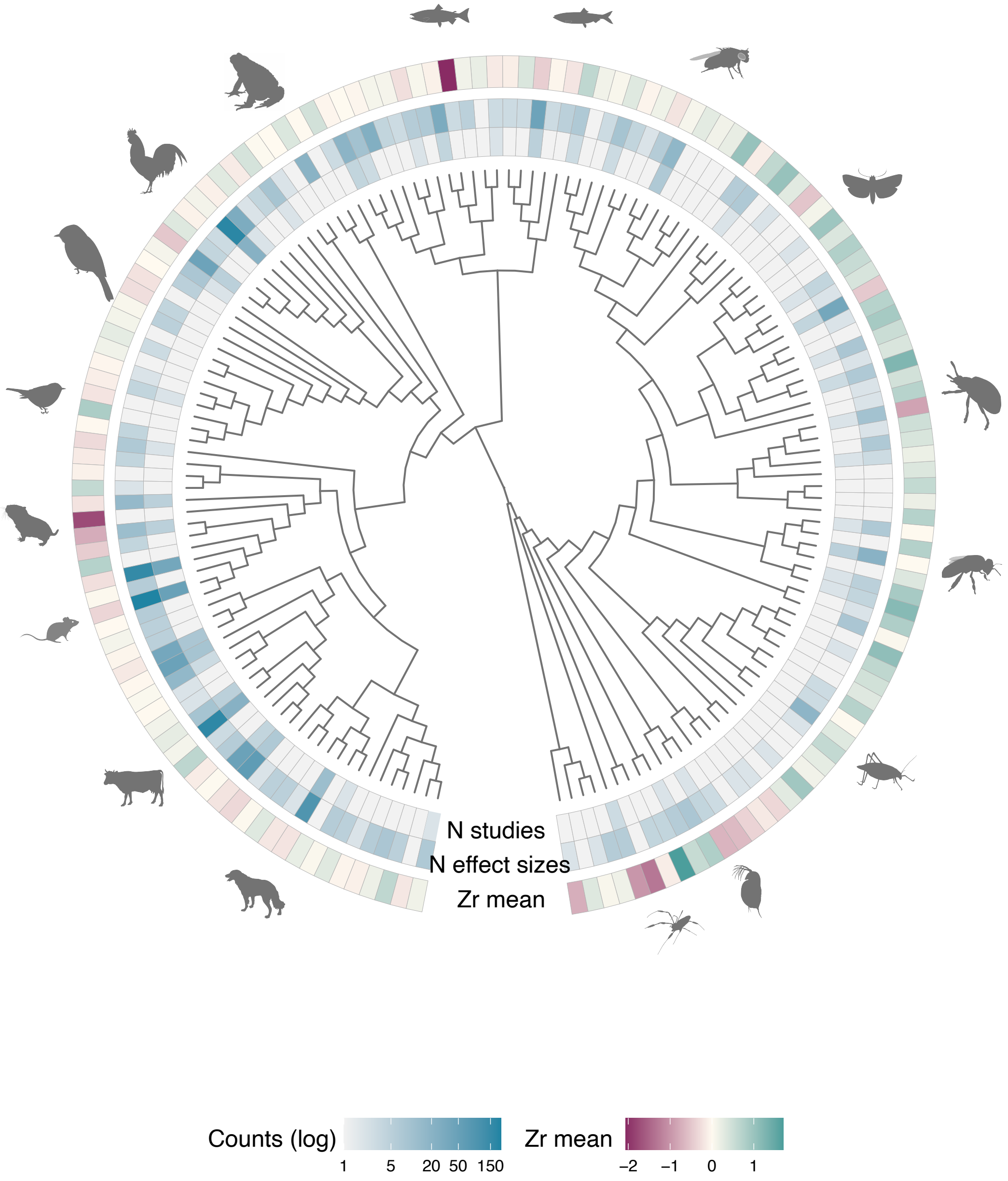

Supplementary Fig.2: Phylogenetic relatedness explained significant variance in our data ($I^2 = 35.40\%$). Phylogenetic tree of all species (157) included in our meta-analysis, along with the number (N) of studies and effect sizes represented by each species. Overall mean effect size for each species showed as Fisher's z-transformed correlation coefficient (Zr) with negative values representing senescence with increasing age and positive values representing improvement in ejaculates with increasing age. Species icons from PhyloPic www.phylopic.org, with all associated images under the CC0 1.0 or PDM 1.0 copyright.

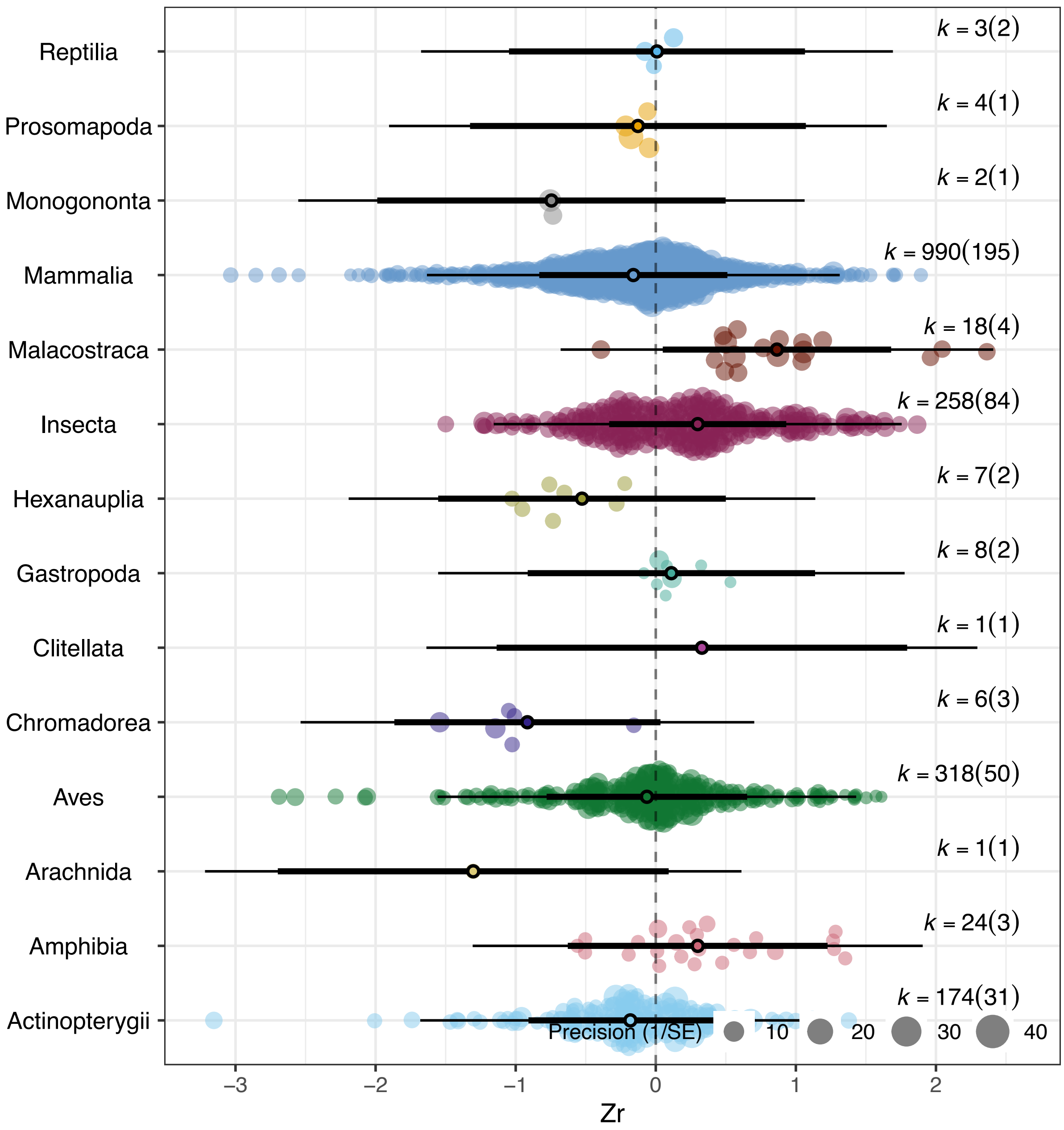

Supplementary Fig.3: Effect of male age on ejaculates for each class. The size of each data point represents the precision of the effect size (1/SE). The X axis represents values of effect sizes as Fisher's z-transformed correlation coefficient (Zr), while the Y axis shows the density distribution of effect sizes. The position of the overall effect is shown by the dark circle, with negative values depicting senescence in ejaculate traits and positive values showing improvement in ejaculate traits with advancing male age. Bold error bars (95% C.I) show whether the overall effect size is significantly different from zero (i.e. not overlapping zero), while light error bars show the 95% prediction interval (P.I.) of effect sizes. Sample sizes reported as: k = number of effect sizes (in brackets: number of studies).

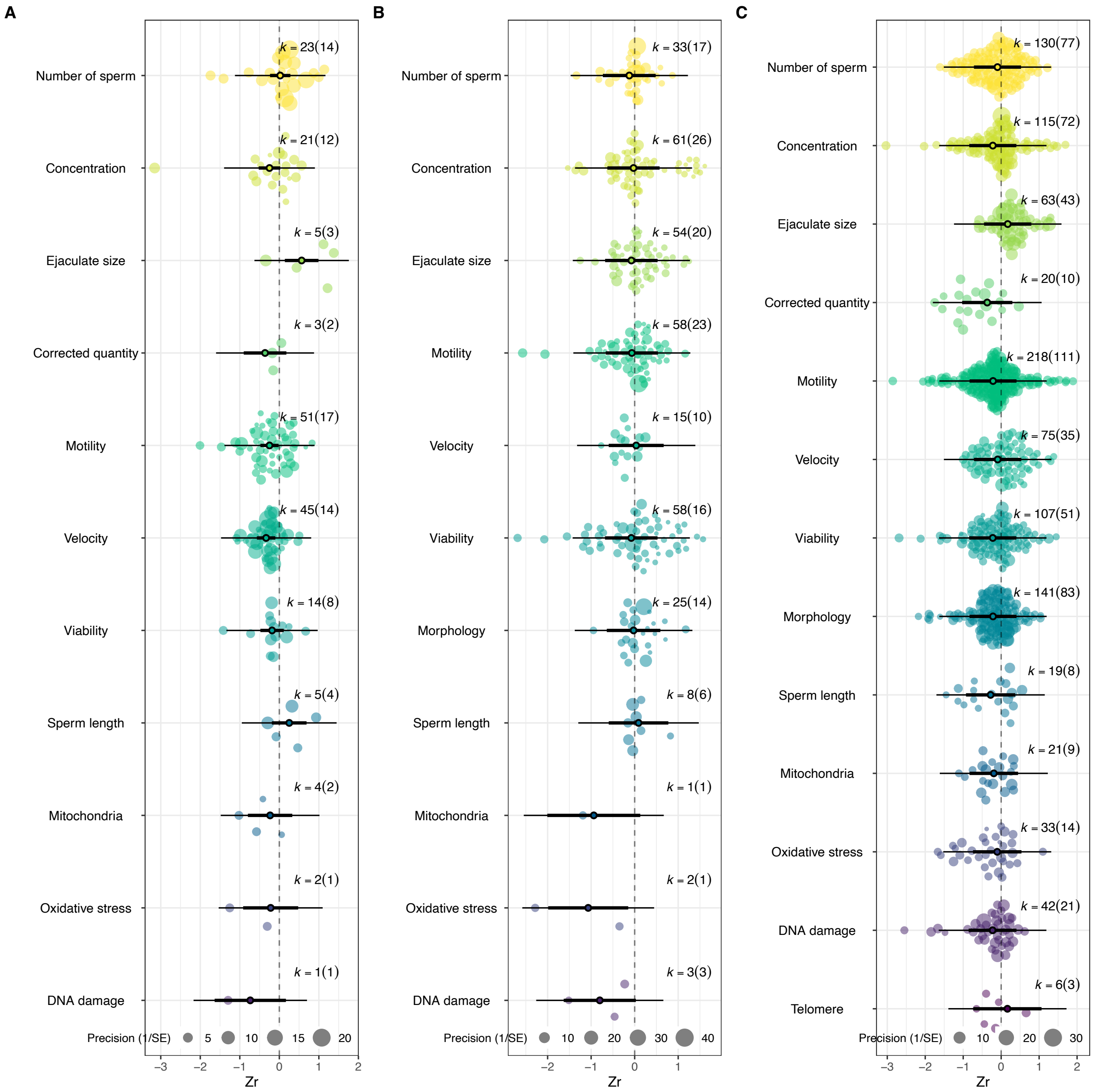

Supplementary Fig.4: Effect of advancing male age on various ejaculate traits for A. Fish, B. Birds, C. Mammals. The size of each data point represents the precision of the effect size (1/SE). The X axis represents values of effect sizes as Fisher's z-transformed correlation coefficient (Zr), while the Y axis shows the density distribution of effect sizes. The position of the overall effect is shown by the dark circle, with negative values depicting senescence in ejaculate traits and positive values showing improvement in ejaculate traits with advancing male age. Bold error bars (95% C.I) show whether the overall effect size is significantly different from zero (i.e. not overlapping zero), while light error bars show the 95% prediction interval (P.I.) of effect sizes. Sample sizes reported as: k = number of effect sizes (in brackets: number of studies).

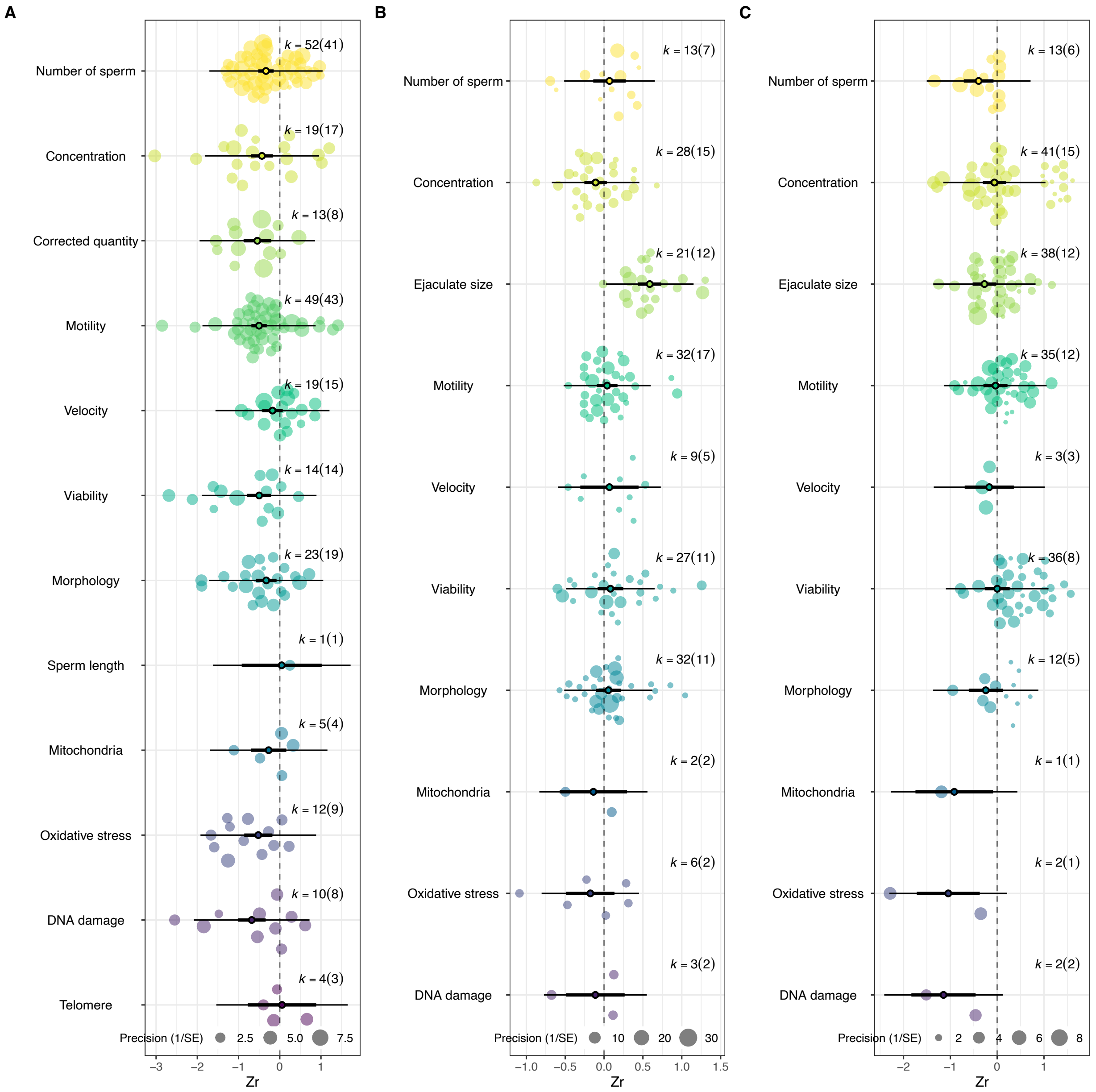

Supplementary Fig.5: Effect of advancing male age on various ejaculate traits for A. Rodents (*Mus musculus* and *Rattus norvegicus*) that did not go through any manipulation. B. Bulls (*Bos taurus*), C. Chickens (*Gallus gallus*). The size of each data point represents the precision of the effect size (1/SE). The X axis represents values of effect sizes as Fisher's z-transformed correlation coefficient (Zr), while the Y axis shows the density distribution of effect sizes. The position of the overall effect is shown by the dark circle, with negative values depicting senescence in ejaculate traits and positive values showing improvement in ejaculate traits with advancing male age. Bold error bars (95% C.I) show whether the overall effect size is significantly different from zero (i.e. not overlapping zero), while light error bars show the 95% prediction interval (P.I.) of effect sizes. Sample sizes reported as: k = number of effect sizes (in brackets: number of studies).

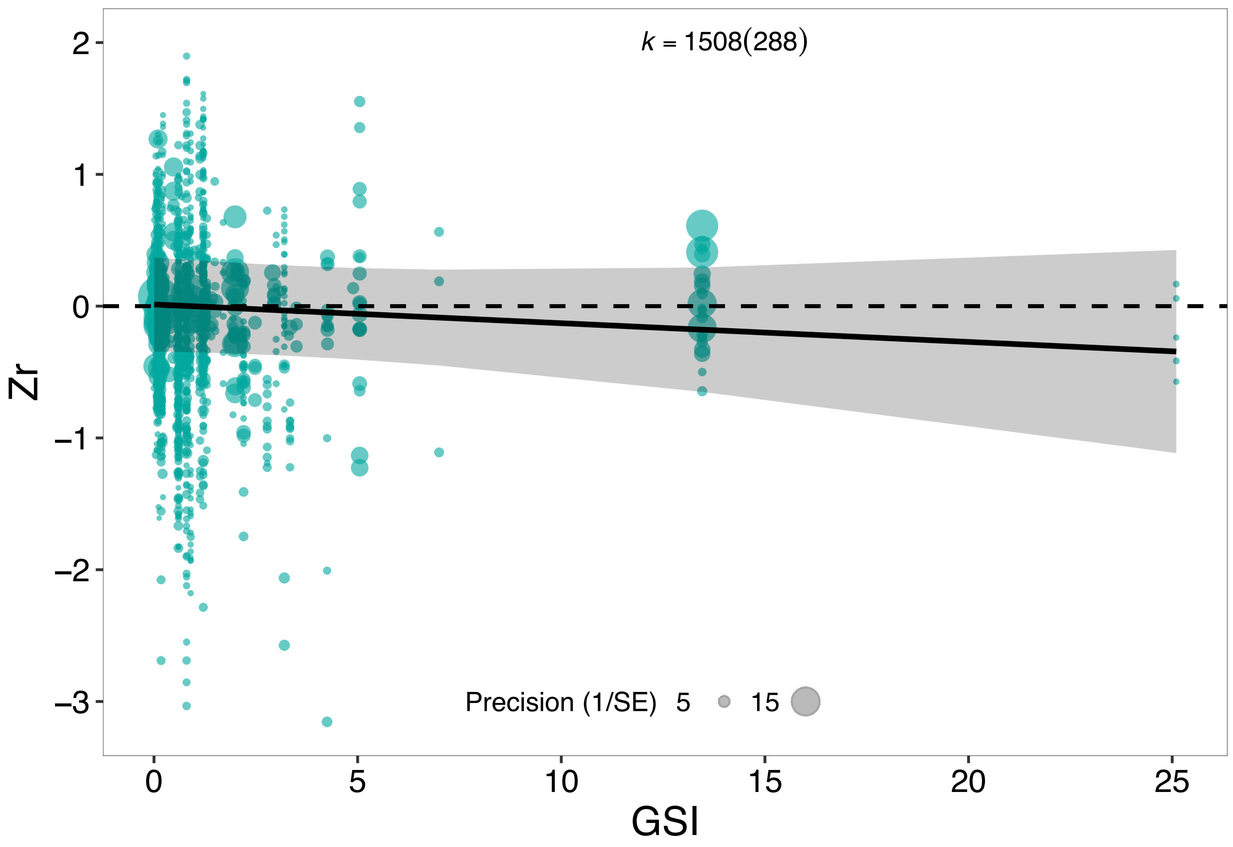

Supplementary Fig.6: Relationship between gonadosomatic index (GSI) and effect sizes for ejaculate senescence. Grey lines indicate 95% C.I.

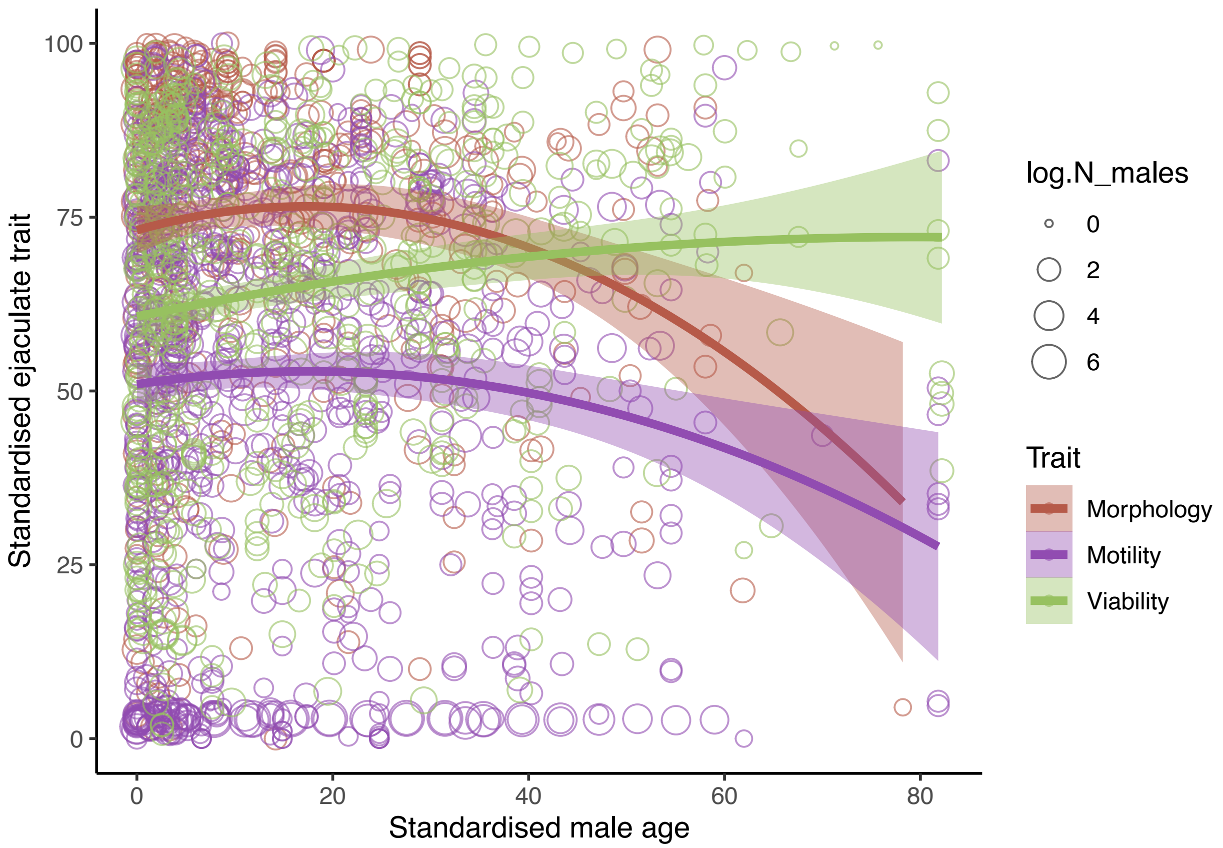

Supplementary Fig.7: Effects of standardised male age (X axis) on standardised ejaculate trait values (Y axis). These are separately shown for % morphologically normal sperm, % motile sperm, and % viable sperm). Standardised male age was calculated as the proportion of maximum adult lifespan represented by the specific age class. Standardised trait value was calculated as percentage of morphologically normal sperm (N= 85 studies, k= 153 effect sizes), percentage of motile sperm (N= 137 studies, k= 294 effect sizes), or percentage of viable sperm (N= 81 studies, k= 193 effect sizes). Shaded lines indicate 95% C.I.

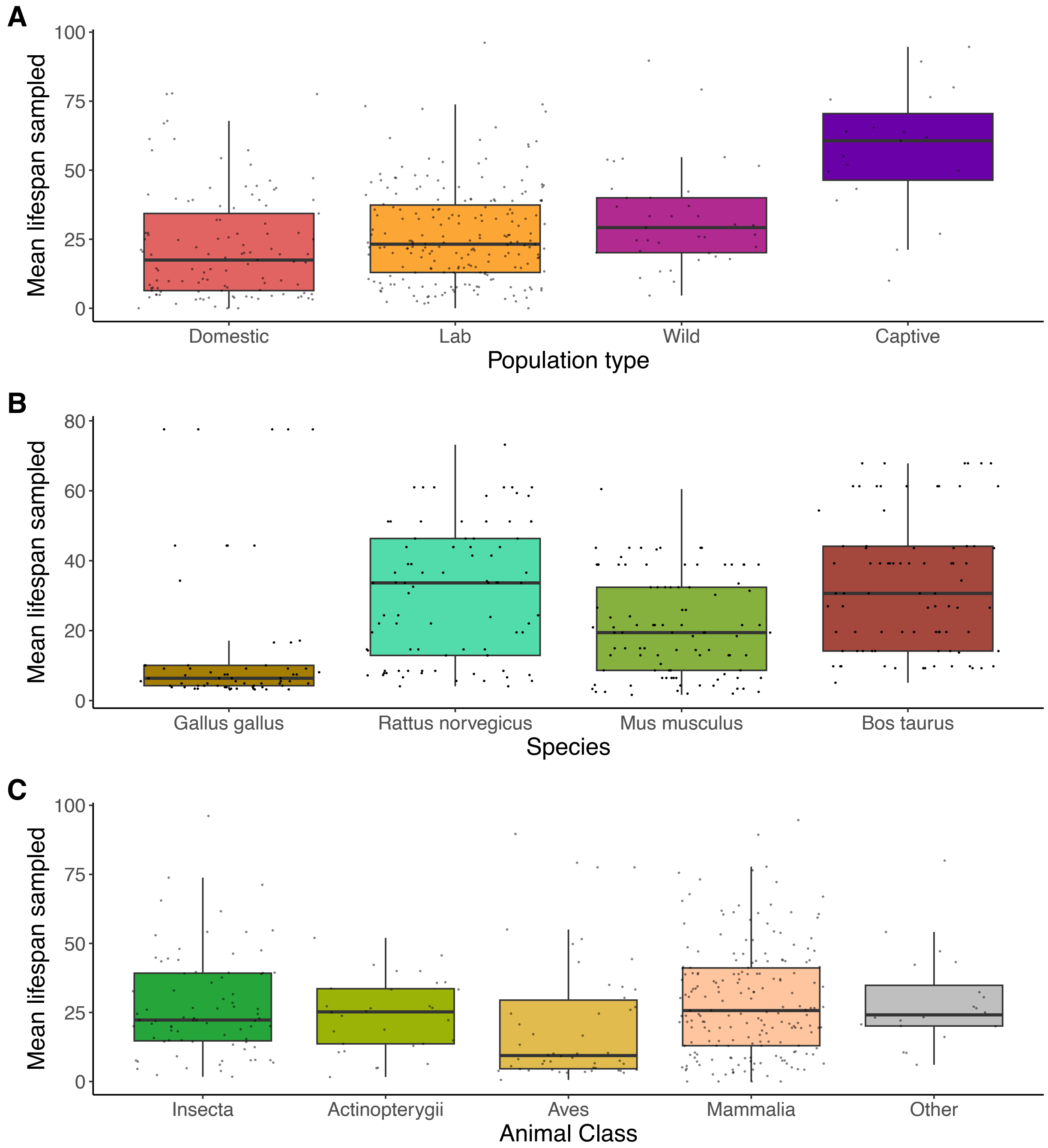

Supplementary Fig 8: Medians (50%) and interquartile ranges (5%, 25%, 75%, 95%) of proportion of maximum adult lifespan sampled for A. Different population types, B. Four most common species in our dataset, and C. Different animal classes (note that animal classes with less than 25 effect sizes were grouped together in 'Other'). Each point depicts the average lifespan sampled from a study.

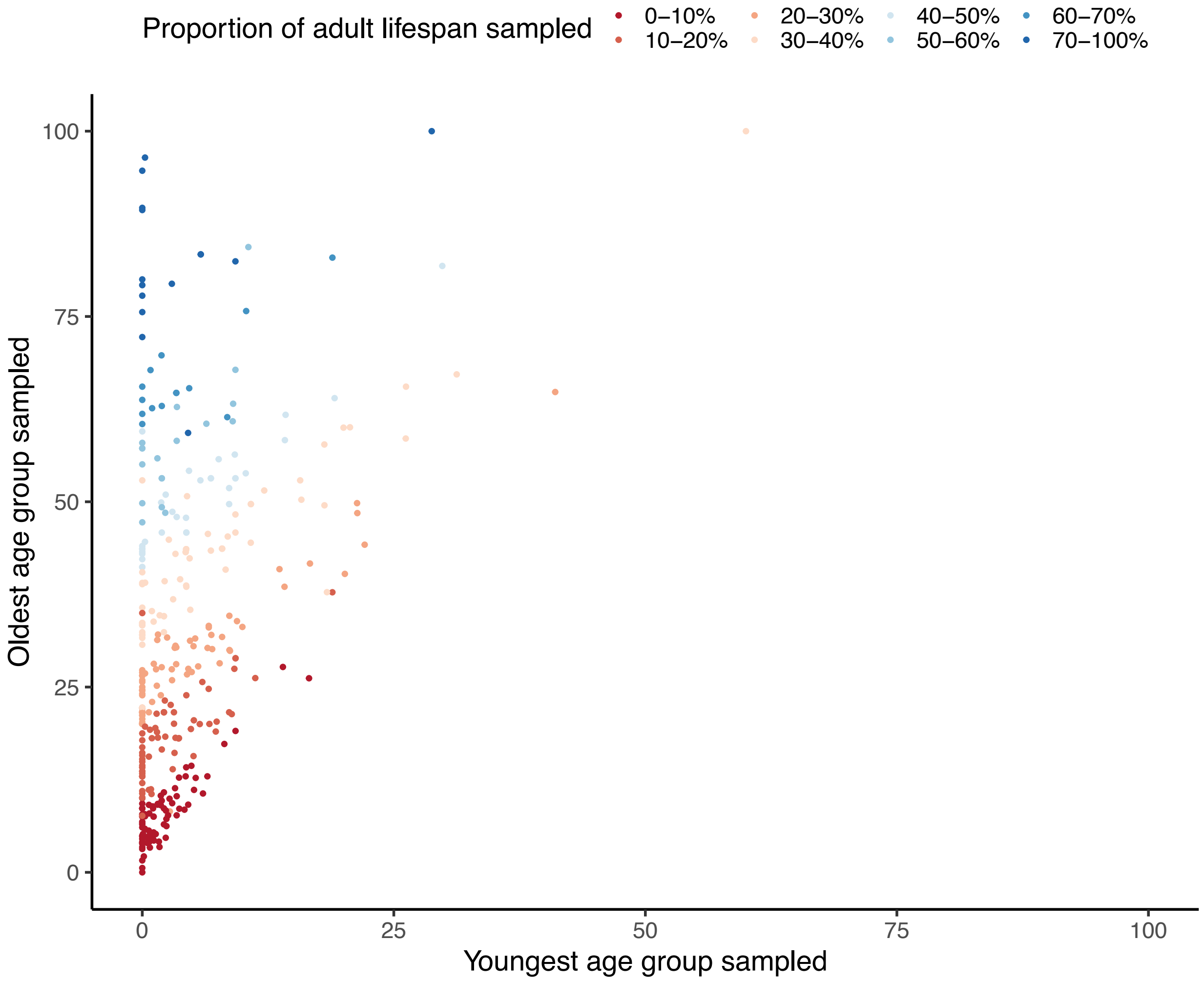

Supplementary Fig.9: Relationship between the youngest and oldest age sampled (as proportion of adult lifespan sampled) for each study (n = 362). Colours indicate the proportion of maximum adult lifespan sampled

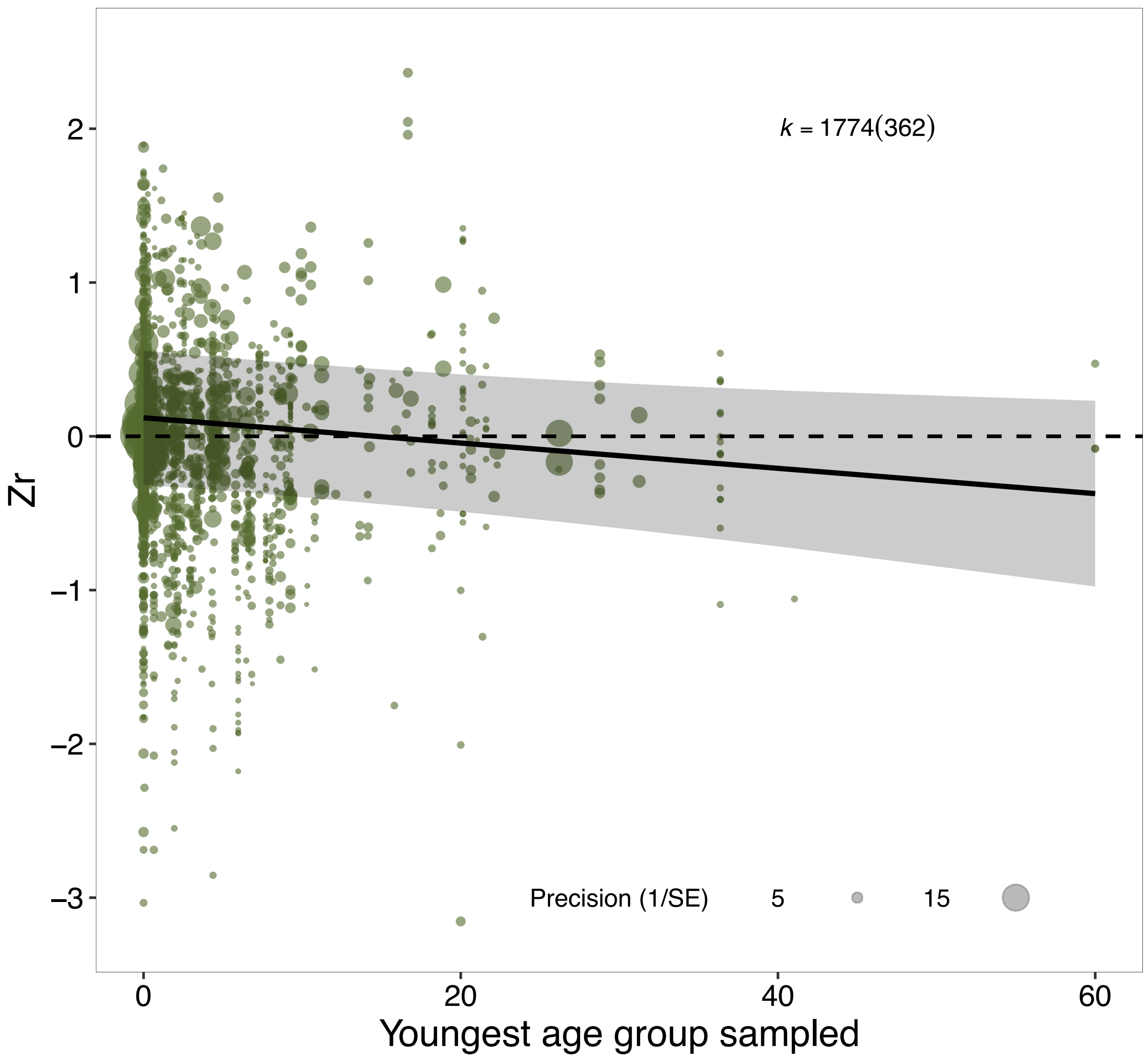

Supplementary Fig.10: Relationship between the youngest age sampled (as proportion of maximum lifespan sampled) and effect sizes for ejaculate senescence

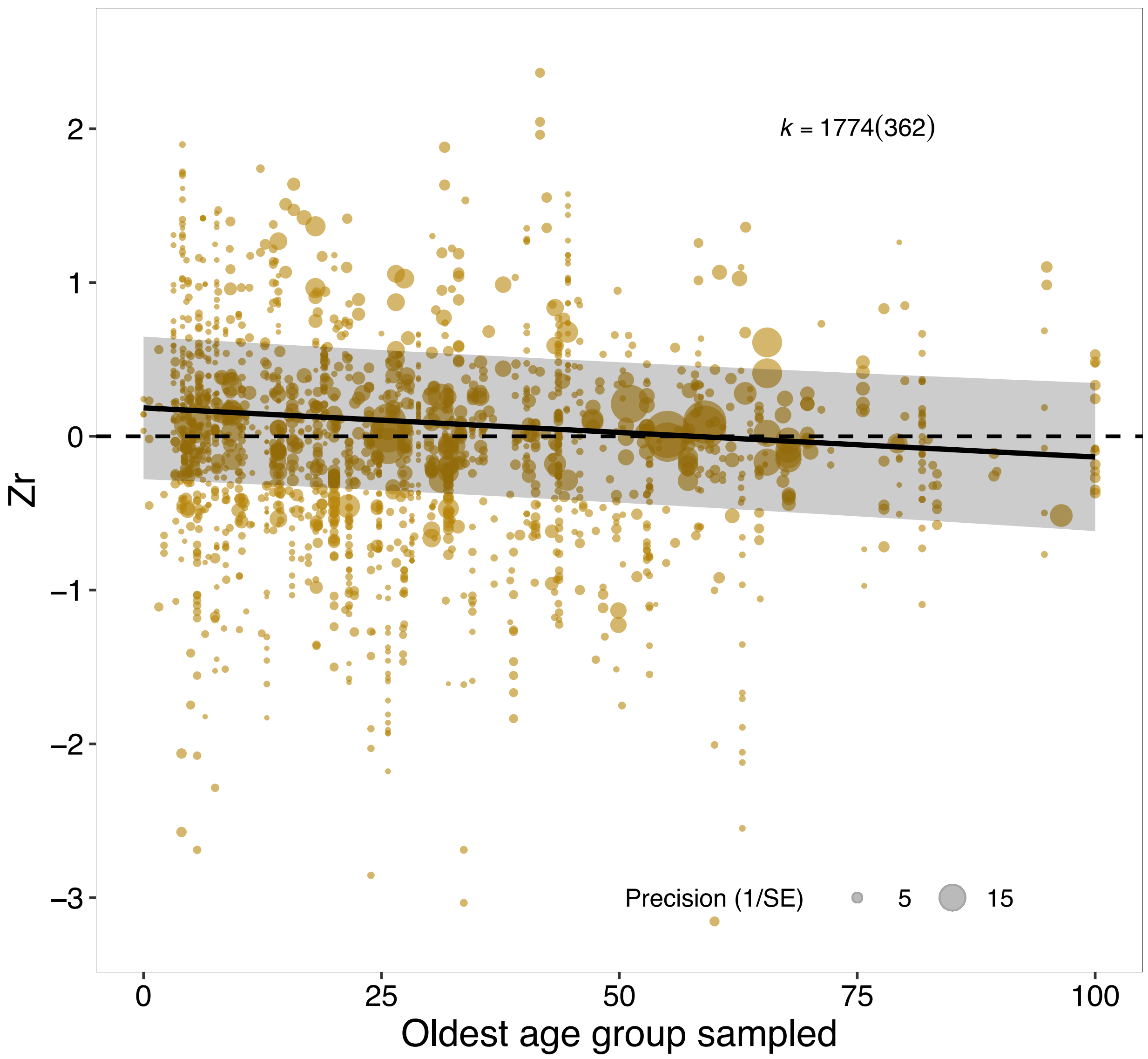

Supplementary Fig.11: Relationship between the oldest age sampled (as proportion of maximum lifespan sampled) and effect sizes for ejaculate senescence

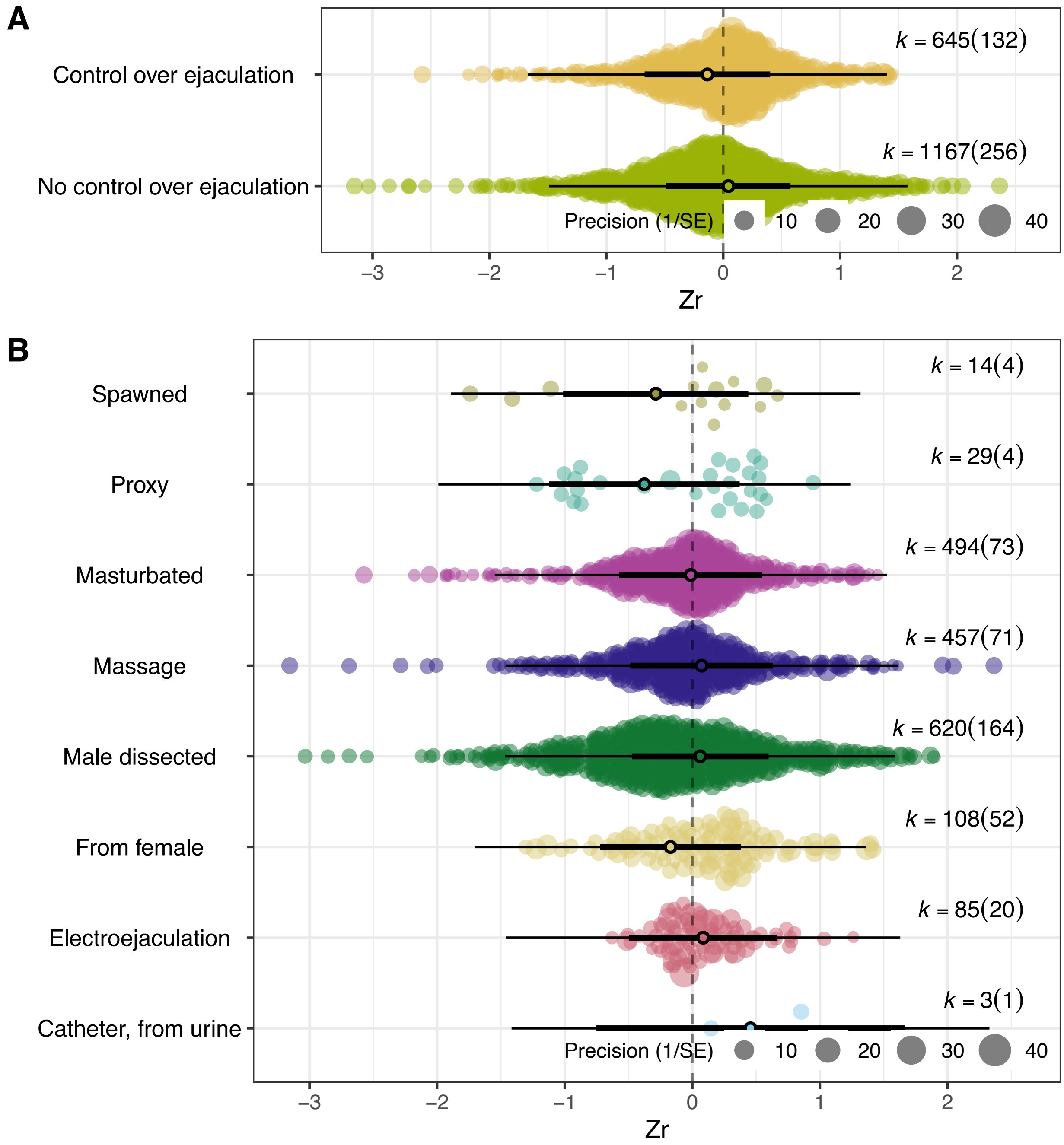

Supplementary Fig.12: A. Effect of male's 'control' over ejaculation on advancing male age. See Supplementary section 7 for definitions. B. Effect of advancing male age on ejaculates for each type of ejaculate collection method. The size of each data point represents the precision of the effect size (1/SE). The X axis represents values of effect sizes as Fisher's z-transformed correlation coefficient (Zr), while the Y axis shows the density distribution of effect sizes. The position of the overall effect is shown by the dark circle, with negative values depicting senescence in ejaculate traits and positive values showing improvement in ejaculate traits with advancing male age. Bold error bars (95% C.I) show whether the overall effect size is significantly different from zero (i.e. not overlapping zero), while light error bars show the 95% prediction interval (P.I.) of effect sizes. Sample sizes reported as: k = number of effect sizes (in brackets: number of studies).

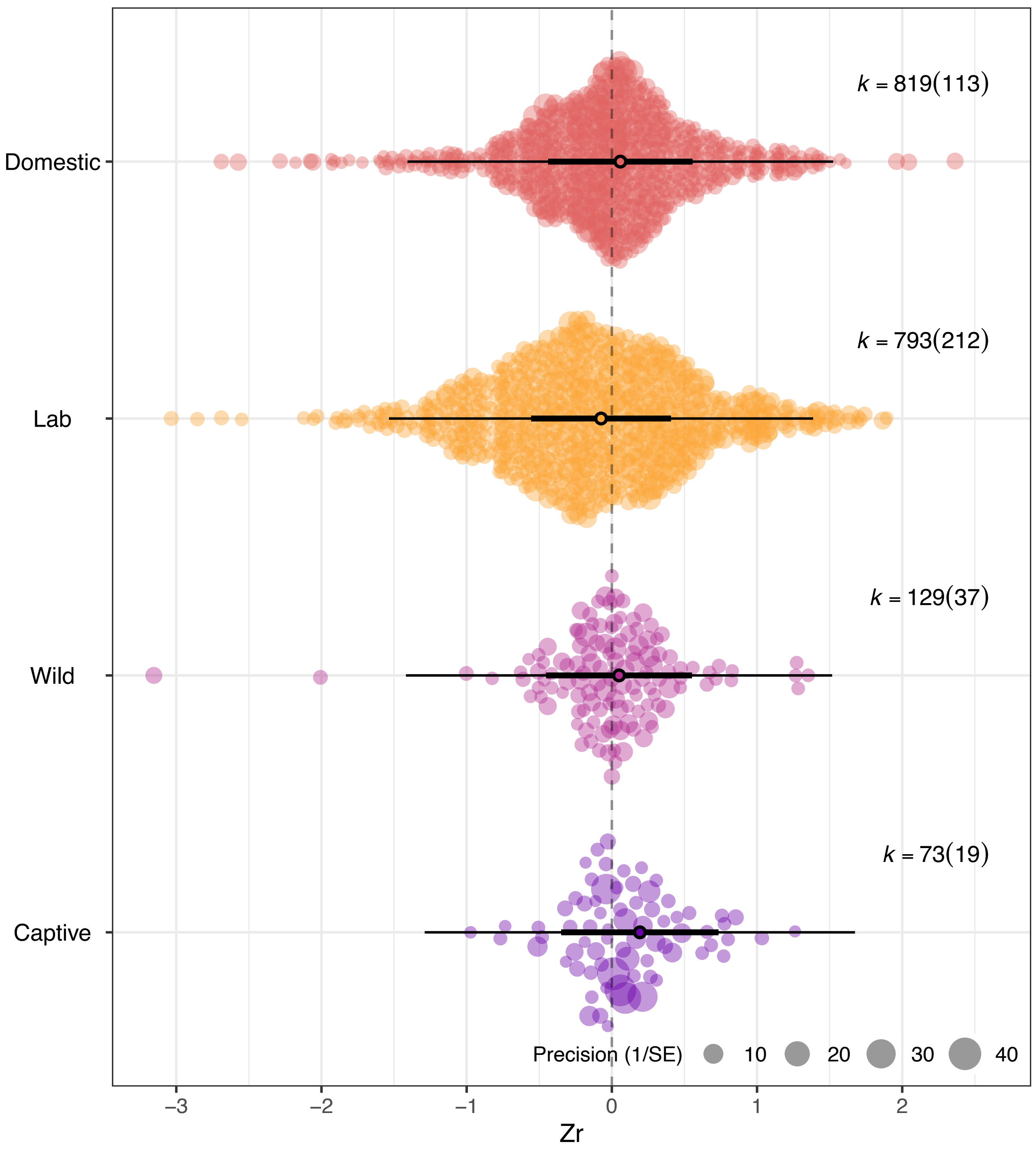

Supplementary Fig.13: Effect of advancing male age on ejaculate traits for different types of population. The size of each data point represents the precision of the effect size (1/SE). The X axis represents values of effect sizes as Fisher's z-transformed correlation coefficient (Zr), while the Y axis shows the density distribution of effect sizes. The position of the overall effect is shown by the dark circle, with negative values depicting senescence in ejaculate traits and positive values showing improvement in ejaculate traits with advancing male age. Bold error bars (95% C.I) show whether the overall effect size is significantly different from zero (i.e. not overlapping zero), while light error bars show the 95% prediction interval (P.I.) of effect sizes. Sample sizes reported as: k = number of effect sizes (in brackets: number of studies).

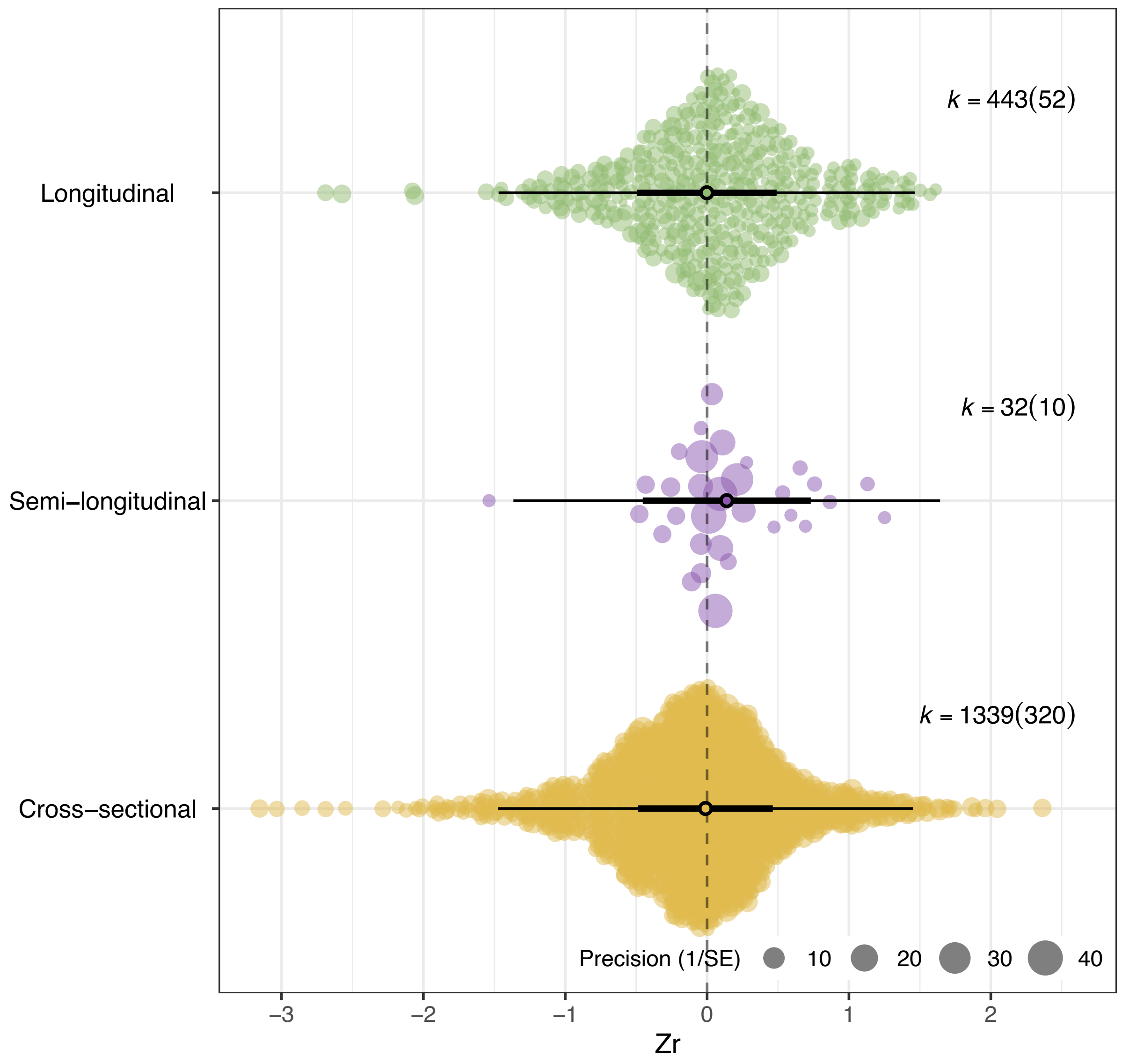

Supplementary Fig.14: Effect of advancing male age on ejaculates for studies with longitudinal vs cross-sectional sampling on males' ejaculates. The size of each data point represents the precision of the effect size (1/SE). The X axis represents values of effect sizes as Fisher's z-transformed correlation coefficient (Zr), while the Y axis shows the density distribution of effect sizes. The position of the overall effect is shown by the dark circle, with negative values depicting senescence in ejaculate traits and positive values showing improvement in ejaculate traits with advancing male age. Bold error bars (95% C.I) show whether the overall effect size is significantly different from zero (i.e. not overlapping zero), while light error bars show the 95% prediction interval (P.I.) of effect sizes. Sample sizes reported as: k = number of effect sizes (in brackets: number of studies).

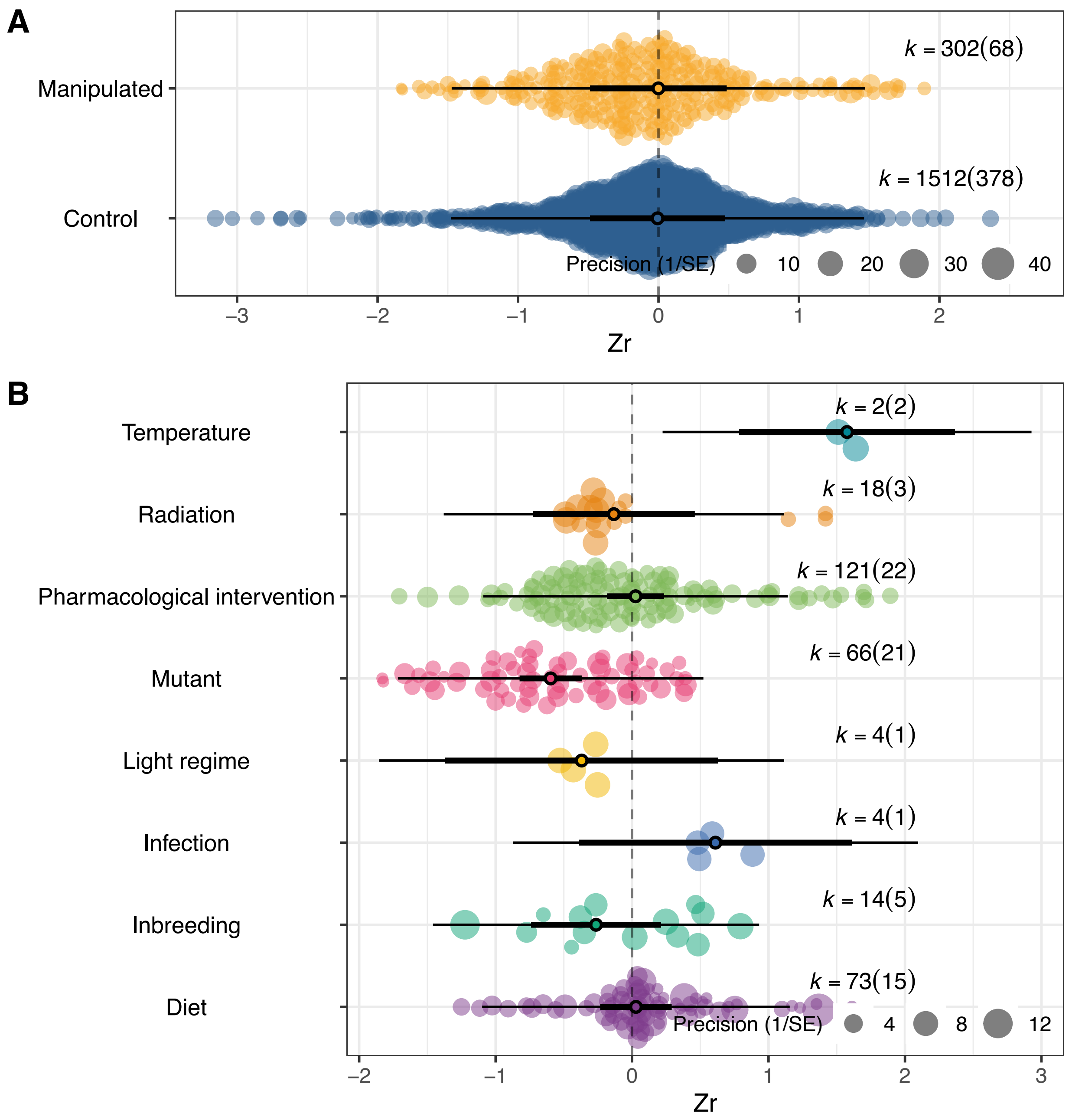

Supplementary Fig.15: A. Effect of advancing male age on ejaculates for studies with 'control' vs 'manipulated' males (see Supplementary section 9 for definitions), B. Effects of advancing male age on ejaculates for each type of manipulation/treatment including manipulated males only. The size of each data point represents the precision of the effect size (1/SE). The X axis represents values of effect sizes as Fisher's z-transformed correlation coefficient (Zr), while the Y axis shows the density distribution of effect sizes. The position of the overall effect is shown by the dark circle, with negative values depicting senescence in ejaculate traits and positive values showing improvement in ejaculate traits with advancing male age. Bold error bars (95% C.I) show whether the overall effect size is significantly different from zero (i.e. not overlapping zero), while light error bars show the 95% prediction interval (P.I.) of effect sizes. Sample sizes reported as: k = number of effect sizes (in brackets: number of studies).

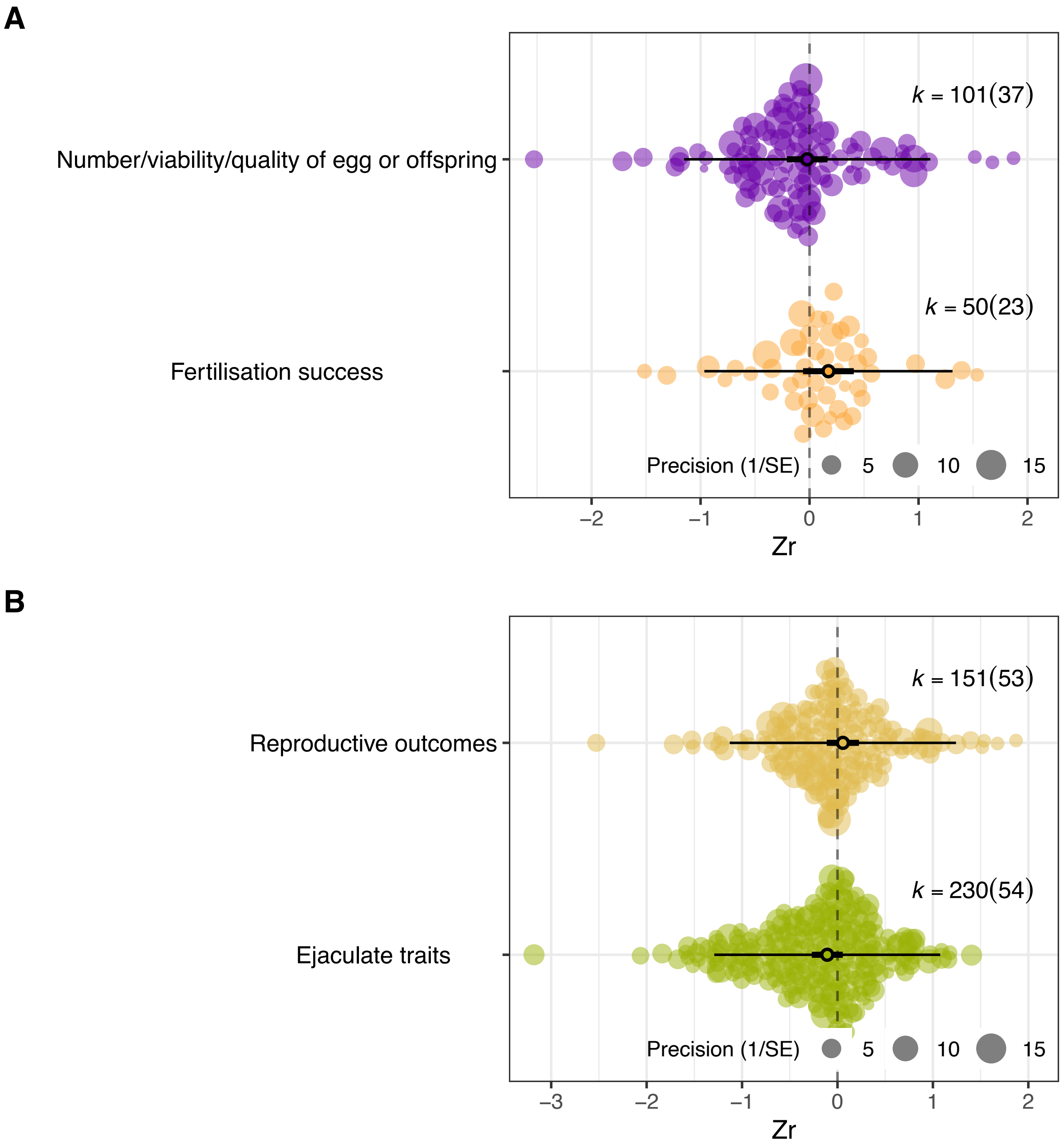

Supplementary Fig.16: A. Effect of advancing male age on the type of fitness trait measured in unmanipulated males. B. Effects of advancing male age on reproductive output and on ejaculates from studies which measure both traits. See Supplementary section 9 for definitions. The X axis represents values of effect sizes as Fisher's z-transformed correlation coefficient (Zr), while the Y axis shows the density distribution of effect sizes. The position of the overall effect is shown by the dark circle, with negative values depicting senescence in ejaculate traits and positive values showing improvement in ejaculate traits with advancing male age. Bold error bars (95% C.I) show whether the overall effect size is significantly different from zero (i.e. not overlapping zero), while light error bars show the 95% prediction interval (P.I.) of effect sizes. Sample sizes reported as: k = number of effect sizes (in brackets: number of studies).

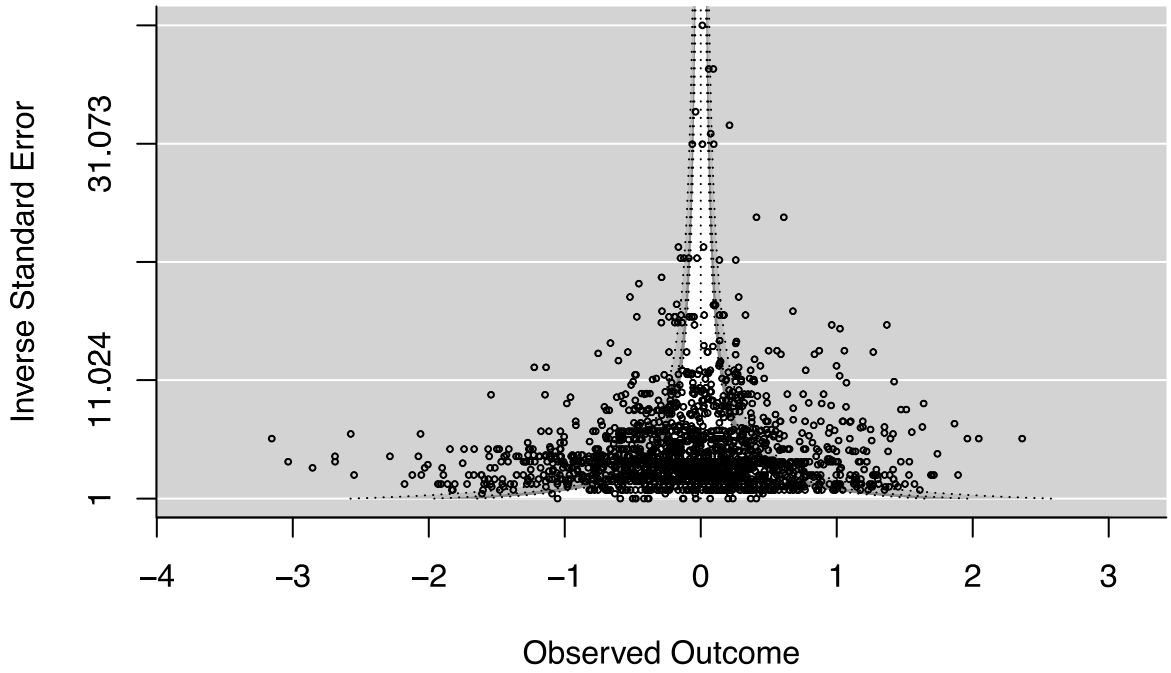

Supplementary Fig.17: Funnel plot of the precision (1/SE) and the residual effect (Observed outcome) from the null model to explore the existence of outliers in the dataset.

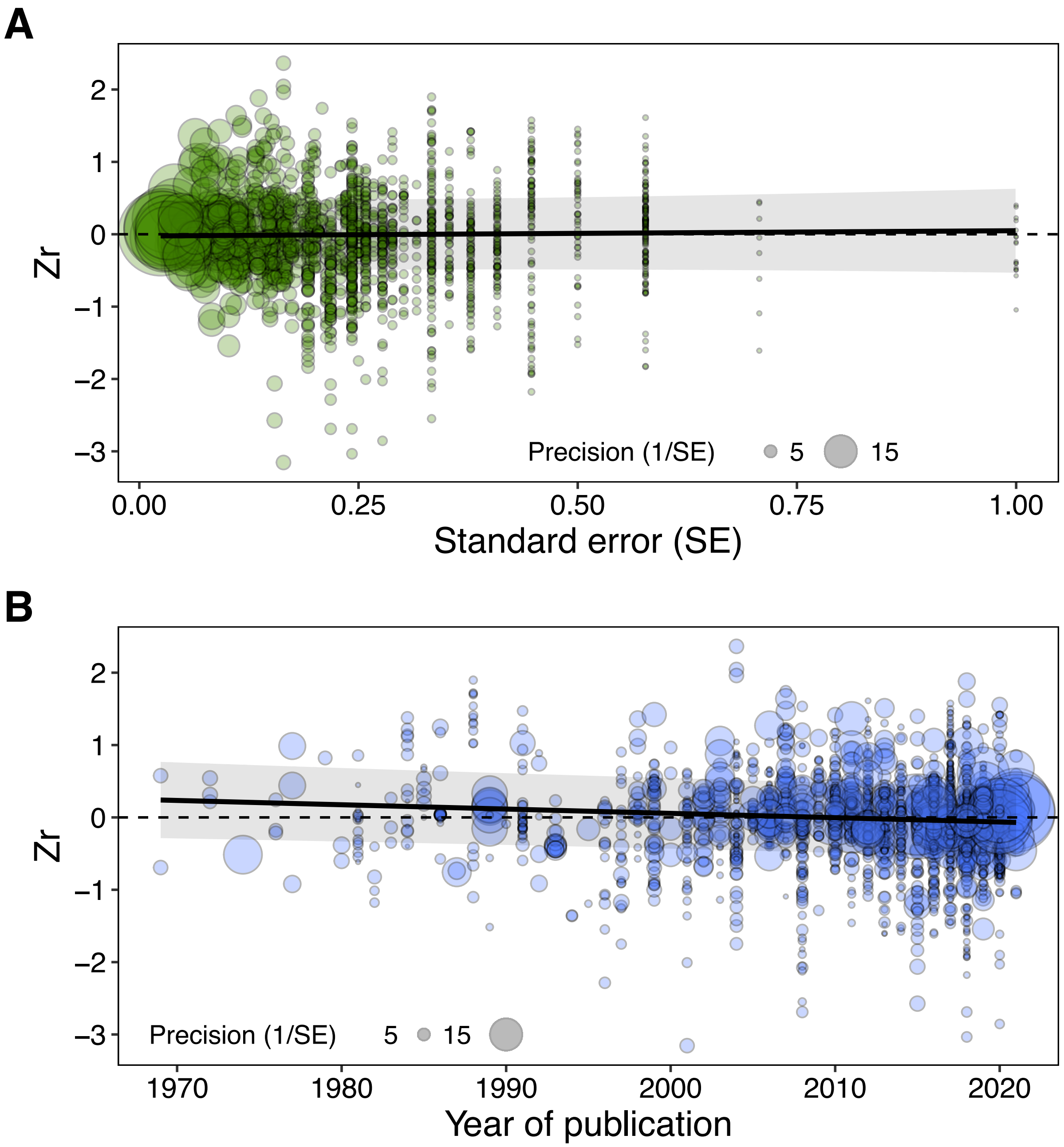

Supplementary Fig.18: Relationship between A. Standard error and effect size estimates (Zr), and B. Year of publication and effect size estimates (Zr). The points are scaled according to the inverse of their variance, so that larger points are given greater weight in the model and represent more reliable estimates. Shaded lines indicate 95% C.I.

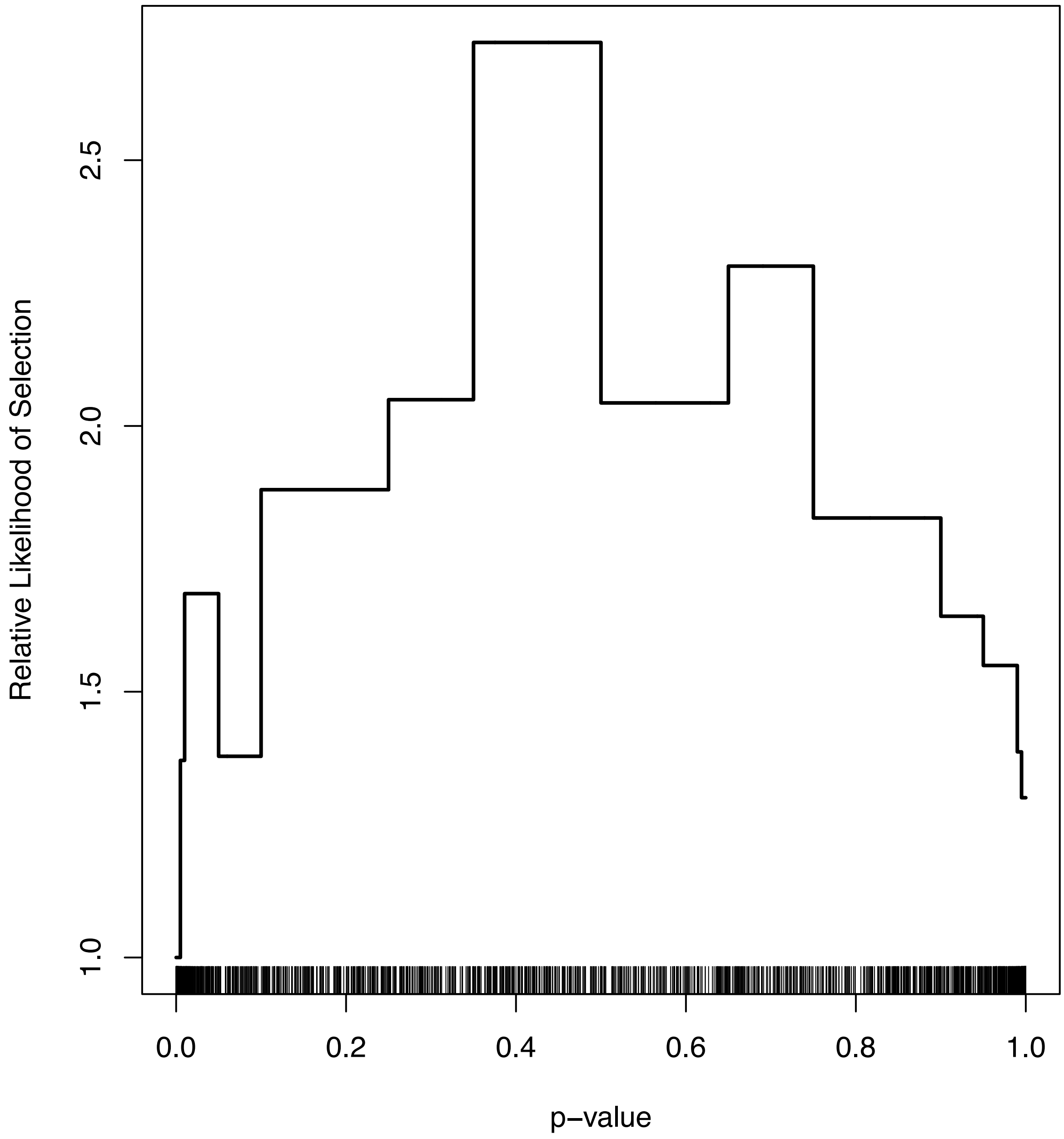

Supplementary Fig.19: Results of a step function selection model based on several cut-points for a model without any moderators.

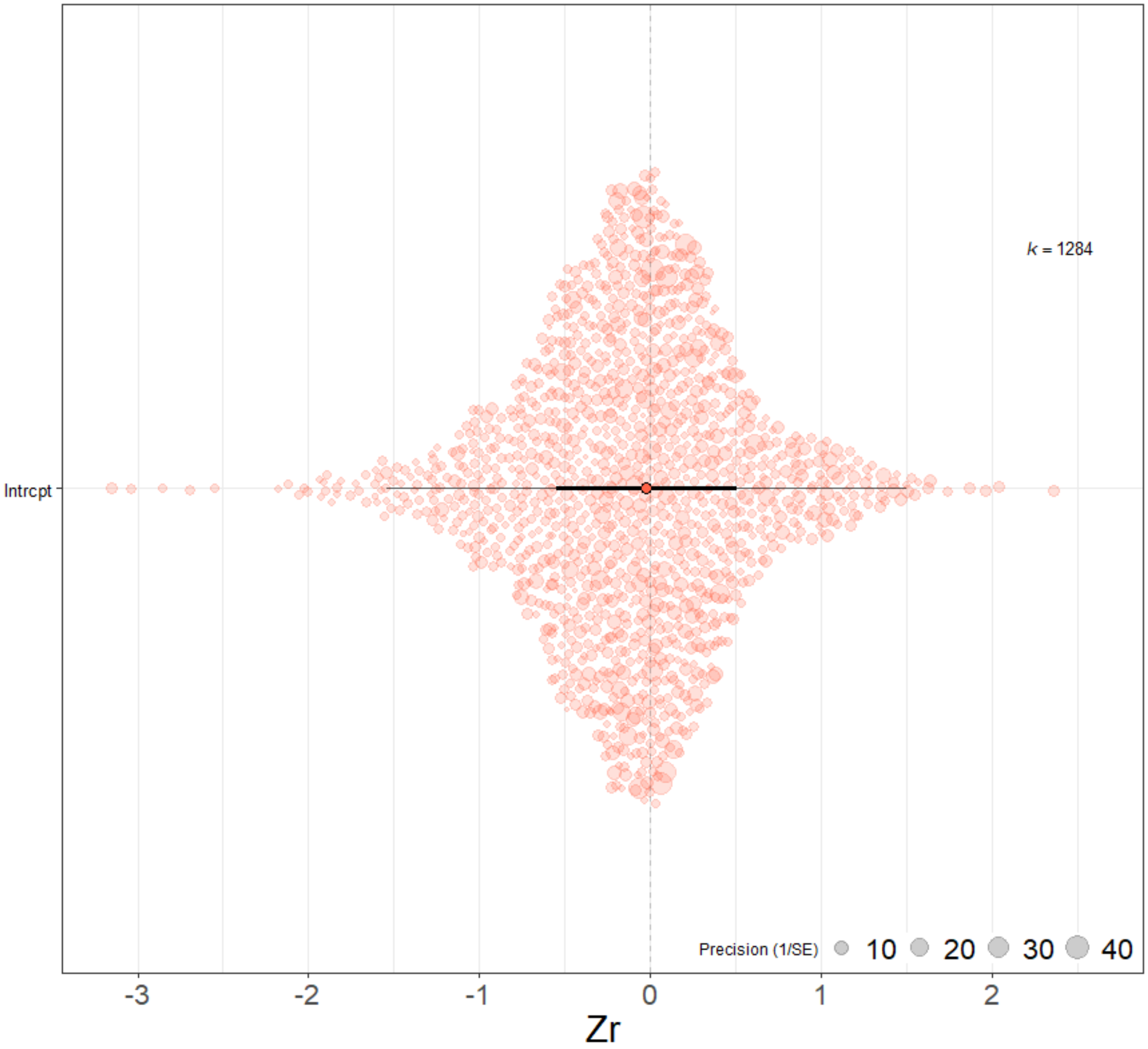

Supplementary Fig.20: The overall effect of advancing male age on ejaculates for studies with >10% of lifespan sampled

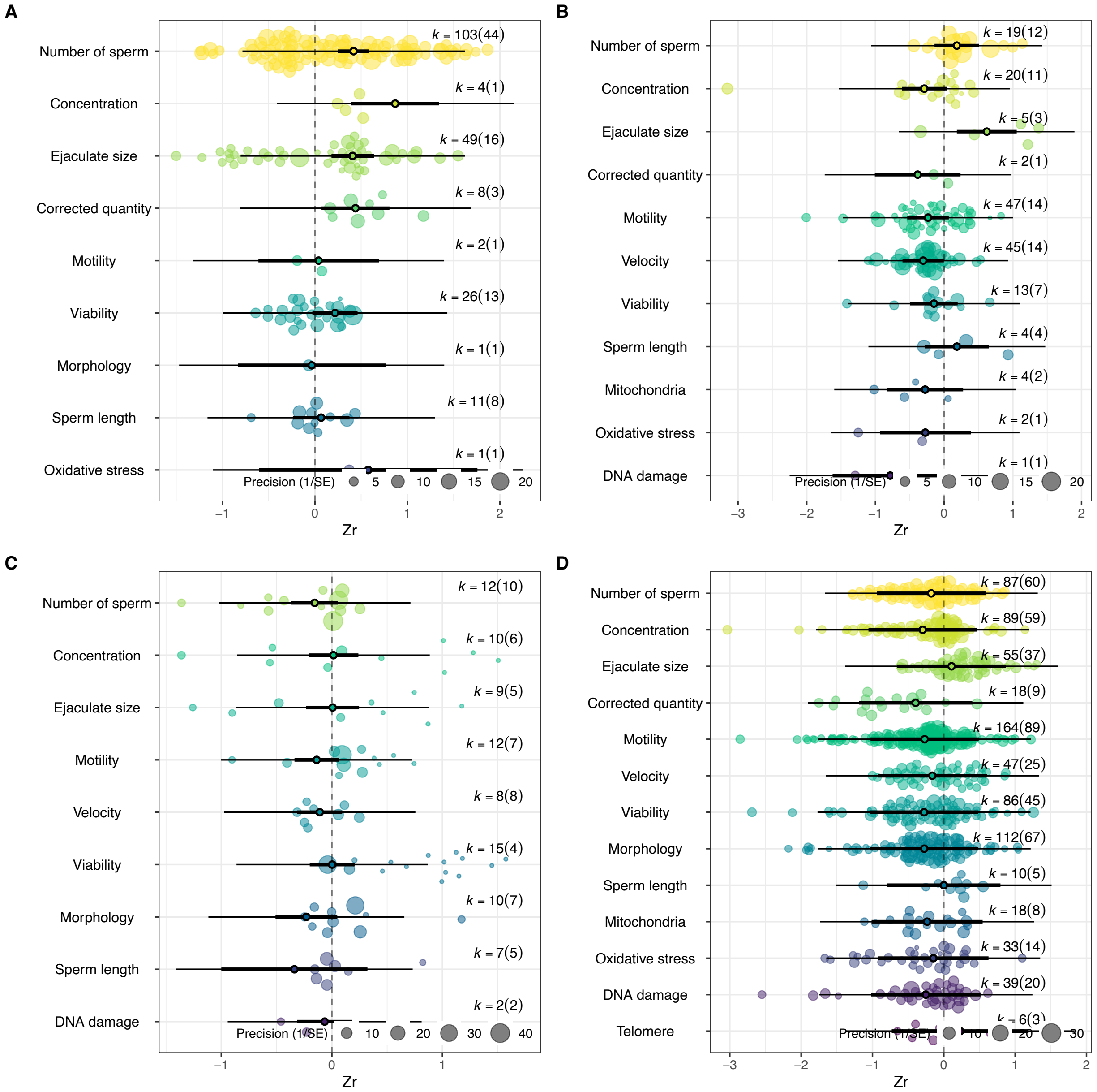

Supplementary Fig.21: Effect of advancing male age on various ejaculate traits when >10% of lifespan was sampled for A. Insects, B. Fish, C. Birds, D. Mammals. The size of each data point represents the precision of the effect size (1/SE). The X axis represents values of effect sizes as Fisher's z-transformed correlation coefficient (Zr), while the Y axis shows the density distribution of effect sizes. The position of the overall effect is shown by the dark circle, with negative values depicting senescence in ejaculate traits and positive values showing improvement in ejaculate traits with advancing male age. Bold error bars (95% C.I) show whether the overall effect size is significantly different from zero (i.e. not overlapping zero), while light error bars show the 95% prediction interval (P.I.) of effect sizes. Sample sizes reported as: k = number of effect sizes (in brackets: number of studies).

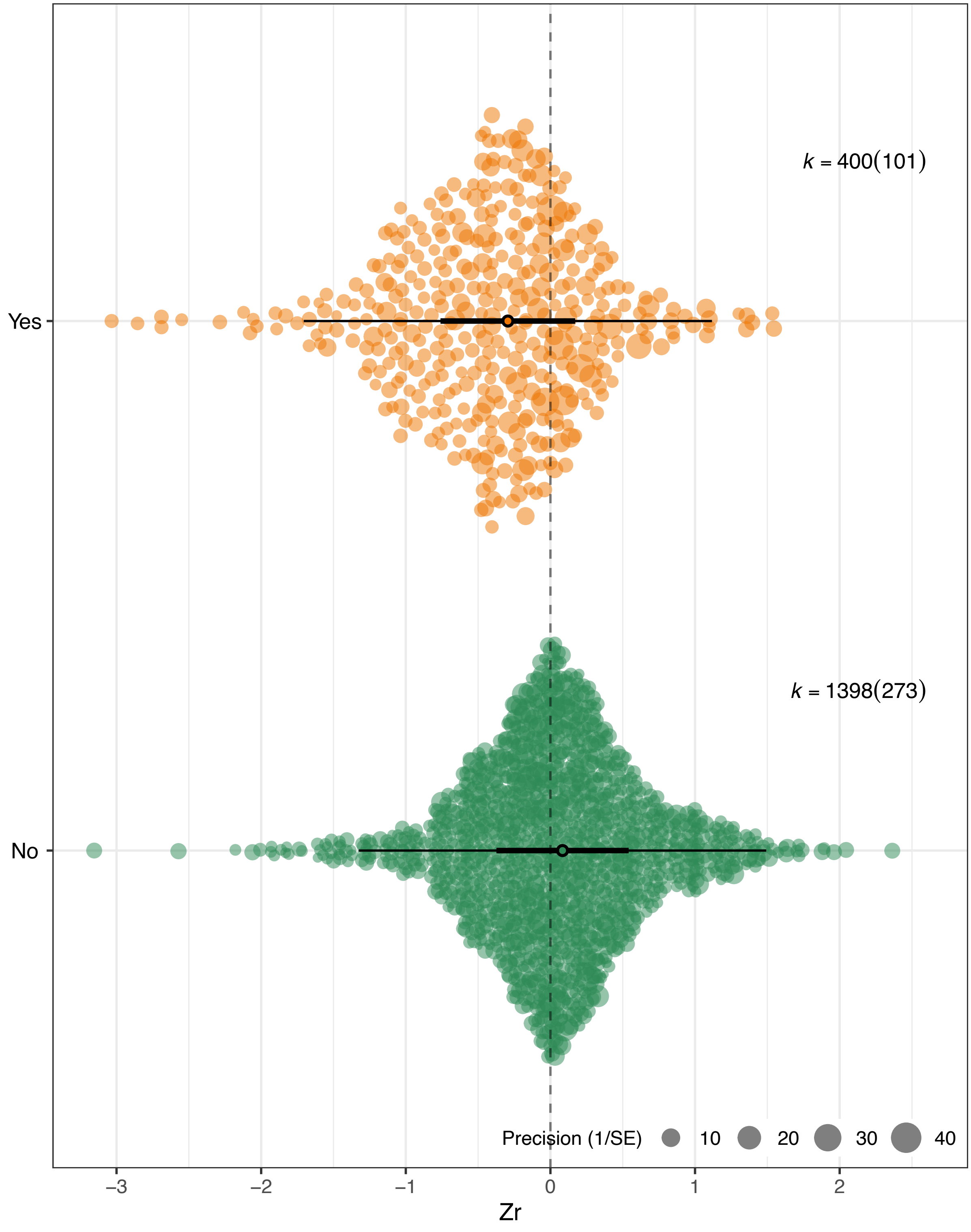

Supplementary Fig.22: Effect of advancing male age on ejaculates when studies explicitly test for an effect of ageing (Yes; i.e. - studies that used the words - ageing, aging, senescence, senescent, or senescing in their abstracts or titles and determined to be interested in senescence) vs studies that do not explicitly test for senescence (No).

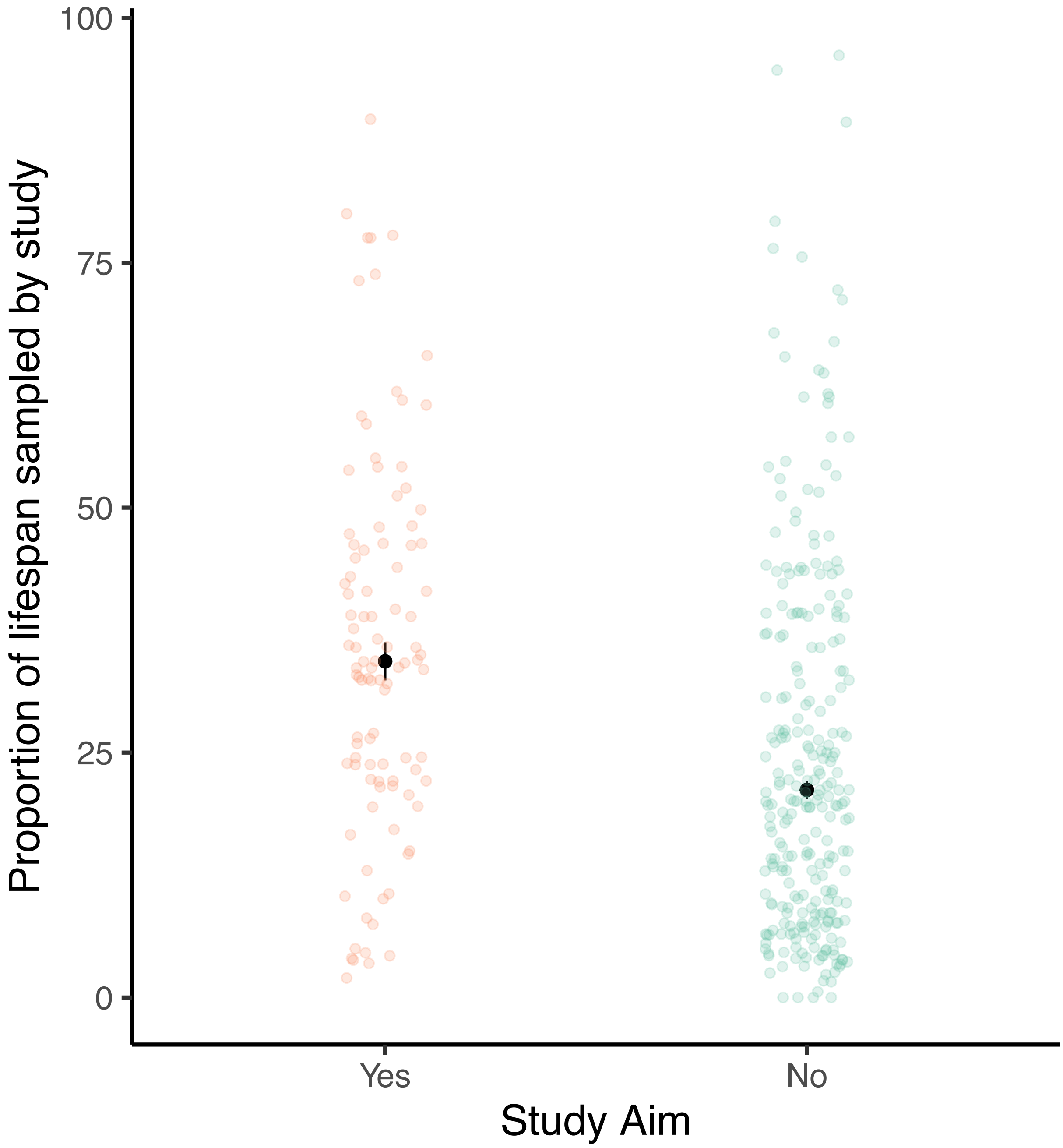

Supplementary Fig.23: Means and 95% CI of lifespan sampled for studies that explicitly test for senescence (Yes; i.e. - studies that used the words - ageing, aging, senescence, senescent, or senescing in their abstracts or titles and determined to be interested in senescence) vs studies that do not explicitly test for senescence (No). Each point refers to the average lifespan sampled from a study.

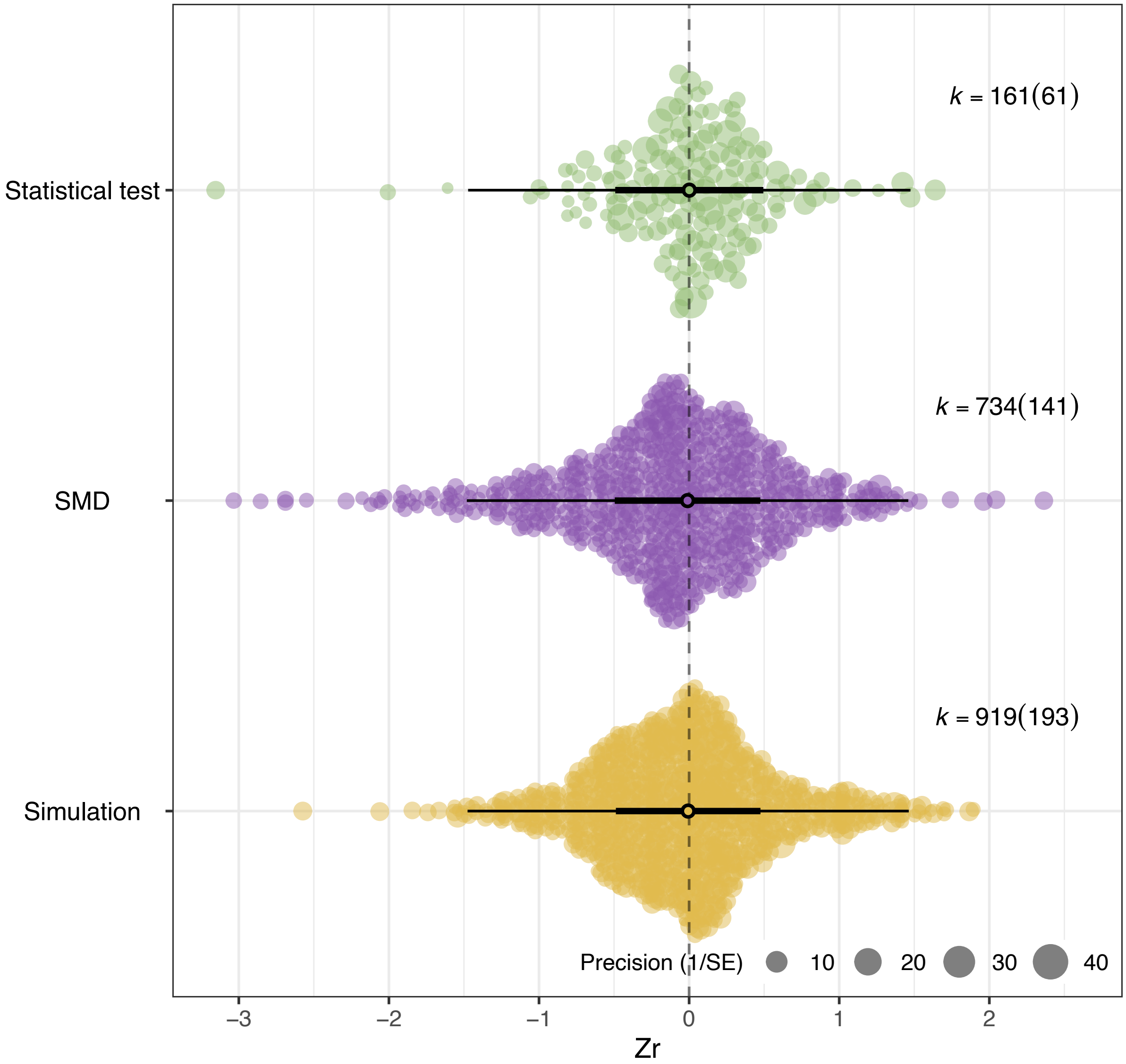

Supplementary Fig.24: Effect of method used to calculate effect sizes. Effect sizes were calculated from statistical tests (Test stat), standardized mean difference (SMD - when only two age groups were available) or through a simulation (when more than two age groups were available). The size of each data point represents the precision of the effect size (1/SE). The X axis represents values of effect sizes as Fisher's z-transformed correlation coefficient (Zr), while the Y axis shows the density distribution of effect sizes. The position of the overall effect is shown by the dark circle, with negative values depicting senescence in ejaculate traits and positive values showing improvement in ejaculate traits with advancing male age. Bold error bars (95% C.I) show whether the overall effect size is significantly different from zero (i.e. not overlapping zero), while light error bars show the 95% prediction interval (P.I.) of effect sizes. Sample sizes reported as: k = number of effect sizes (in brackets: number of studies).

Supplementary notes

Supplementary notes 1: Search string

We first conducted a scoping search on Google Scholar. Our scoping search was done using the keywords "male age sperm -human", and we used the first 48 papers (i.e. all papers from the first 5 pages out of 11800 pages of the search) to build a word cloud to discern the most common words used in these studies. We used the most commonly occurring relevant words from this word cloud to create keywords for our search string.

The table below shows the most frequent words from our scoping search collected from 48 relevant abstracts generated through a word cloud. Words used in our search string are highlighted in bold and italics. The selection of words was made such that they were not specific to a single species, nor biologically broad.

frequency	word	frequency	word
**305**	***sperm***	25	size
**191**	***male***	24	fluid
**151**	***age***	24	transfer
104	mating	22	fitness
75	reproductive	21	offspring
66	female	21	proteins
60	females	20	competitive
58	success	20	ejaculates
53	competition	20	fertilization
**47**	***ejaculate***	20	mated
**45**	***old***	20	selection
**45**	***young***	20	transferred
41	quality	19	history
36	traits	19	Sperm
33	older	18	viability
32	seminal	17	associated
31	species	17	rate
30	***ageing***	16	Drosophila
29	fertility	16	paternity
29	sexual	16	production
**28**	***senescence***	**16**	***semen***
27	mate	**14**	***aging***
25	ability	14	cells

Distinct Boolean characters used for the search strings in each database.

<u>Search string used for SCOPUS:</u>

"(TITLE-ABS-KEY (male) AND TITLE-ABS-KEY (age) OR TITLE-ABS-KEY (ageing) OR TITLE-ABS-KEY (aging) OR TITLE-ABS-KEY (senesc*) OR TITLE-ABS-KEY (old) OR TITLE-ABS-KEY (young) AND TITLE-ABS-KEY (sperm) OR TITLE-ABS-KEY (ejaculate) OR TITLE-ABS-KEY (semen) AND NOT TITLE-ABS-KEY (human) OR TITLE-ABS-KEY (men)) "

Search string used for Web of Science:

“AB= (male AND (age OR ageing OR senesc* OR aging OR old OR young) AND (sperm OR ejaculate OR semen) NOT (human OR men)) OR TI= (male AND (age OR ageing OR senesc* OR aging OR old OR young) AND (sperm OR ejaculate OR semen) NOT (human OR men))”

Search string used for BASE:

“male age sperm”

Supplementary notes 2: Repeatability

Screening process

To ensure repeatability of the screening process, two analysts screened abstracts and full-texts independently and checked for agreement on included versus excluded studies. Analyst 1 screened all abstracts to check the suitability of each study (n = 9683) while analyst 2 checked for repeatability by screening ~50% of abstracts (n = 4918). Analyst 1 and 2 agreed on the inclusion or exclusion (i.e. suitability) of 91% of the studies when screening abstracts (Kappa = 0.56; Kappa calculates inter-rater reliability for qualitative items, Koricheva et al, 2013). Analyst 1 then screened all full texts that passed the abstract screening stage (n = 1003 studies from published sources and four from unpublished sources) to determine whether a study was suitable to be included in the meta-analysis, while analyst 2 screened ~10% of full texts to test for repeatability (n = 100). Analyst 1 and 2 agreed on the inclusion or exclusion of 98% of the studies when screening full-texts (Kappa = 0.96).

Data extraction

To assure repeatability of data extraction, two analysts checked data obtained from ~5% of papers (*i.e.* 19 studies and 75 effect sizes). To do this, analyst 1 and 2 first independently extracted data and calculated effect sizes (Fisher's z-transformed correlation coefficient) for each of the 75 rows of the data collected. Then a coefficient of determination between the effect sizes obtained by analyst 1 and 2 was calculated ($R^2 = 0.96$, $P<0.001$), indicating strong repeatability.

Supplementary notes 3: Definition of adult

Groups that were described as "[pre-] pubertal", "adolescent", "juvenile", or "immature" in the study were not considered as adults and thus not included in the analysis, because we were only interested in age-dependent changes in adult individuals. For arthropods, we included all post-eclosion/last-moult ages in the analysis because this is when arthropods are considered adults[1]. If all age groups in a study had immature (non-adult) males, we excluded these studies. On the other hand, if at least two age groups of males were adults, we included the study, and we used only the data from adult males.

Supplementary notes 4: calculation of SD

We collected data on means, SD/SE, and sample sizes of males in each age group from: text in the Results section, figures using WebPlotDigitizer[2] and MetaDigitize[3], the supplementary information or raw data provided with the paper, a data repository, or by directly contacting the authors of the paper, in that order. We converted standard errors to standard deviations (SD) using the formula[4] SD = SE* sqrt (N). When medians and inter-quartile ranges were reported, we converted these to SD[5].

Supplementary notes 5: Ejaculate traits and reproductive outcomes definitions

For all studies, we collected data on ejaculate traits as reported by the original paper. When a study reported data for multiple traits, data on all traits were collected. Although, when a study reported data on a whole trait (e.g. % of total motile sperm; % sperm with morphological defects) as well as sub traits (e.g. % progressively motile sperm, % sperm with mid-piece defects), only data from the whole trait was recorded. Due to differences in terminology when describing similar traits between different studies, we created a broad category to describe different types of traits. The categorization of these traits is described below.

Ejaculate traits

**In meta-analysis**	**In paper**
Concentration	Sperm density, concentration, count per mL/ µL, spermatocrit
DNA damage	Sperm chromatin damage, sperm DNA damage, sperm chromatin instability, sperm DNA fragmentation, sperm chromatin structure damage
Ejaculate size	Spermatophore size, ejaculate mass, ejaculate volume, area of seminal vesicle filled with ejaculate
Corrected quantity	Sperm concentration or number as proportion of body mass/testis mass/epididymis mass
Mitochondria	Sperm ATP, sperm metabolic activity, sperm mitochondrial function, sperm mitochondrial activity, sperm mitochondrial membrane potential
Morphology	Normal/abnormal sperm morphology, tail/head/midpiece morphological defects, cytoplasmic droplets on sperm
Motility	Percent progressive motility, percent non-progressive motility, percent total motility, sperm vigor, sperm mass motility, motility (on a numerical subjective scale)
Number of sperm	Number of apyrene sperm, number of eupyrene sperm, number/count of sperm, number of spermatophores, number of sperm bundles
Oxidative stress	Reactive oxygen species in sperm, sperm oxidant or antioxidant levels, sperm lipid peroxidation, sperm glutathione peroxidase, sperm superoxidase dismutase
Sperm length	Total sperm length, sperm tail length
Telomere	Sperm telomere length
Viability	Sperm acrosome integrity, sperm viability, % sperm alive/dead, sperm vitality, sperm membrane integrity

Velocity	Average path velocity (VAP), curvilinear velocity (VCL), straight line velocity (VSL) (in that order of preference, when more than one reported)

Reproductive outcomes

**In meta-analysis**	**In paper**
Fertilization success	Percent eggs fertilized, Percent eggs sired under sperm competition, fertilization rate, fertilization success, PVL hole number, paternity share
Number/viability/quality of egg/offspring	Egg hatchability, egg mass, egg viability, male or female fecundity, hatching success, number of eggs laid, female lifetime reproductive success, number of offspring, number of fetuses, offspring developmental rate, offspring survival, offspring body mass/ size

Supplementary notes 6: Collection of data on sperm competition levels, adult lifespan and age of sexual maturity for different species

We recorded the level of sperm competition faced by a species by collecting data on the Gonadosomatic index (i.e. testis weight as a percentage of body weight), which has been shown to be a reliable predictor of sperm competition. This was done by searching for "testes weight" or "gonadosomatic index" of each species in our dataset on google scholar as well as collecting data from comparative studies on this topic (OSF https://osf.io/dk8sq/ for raw data).

If the study did not report the maximum adult lifespan or age of sexual maturity/adulthood of the species/population being studied, these were collected from other sources, and then used to calculate the percentage of maximum adult lifespan sampled for a given species in the study. Data on maximum adult lifespan of a given species, as well as age of sexual maturity were then used to calculate the proportion of maximum adult lifespan sampled for a given species in a study as follows:

$$Proportion\ of\ maximum\ adult\ lifespan\ sampled = \frac{\left((Max\ age\ sampled - age\ of\ maturity) - (Min\ age\ sampled - age\ of\ maturity)\right)}{(Maximum\ lifespan\ of\ species - age\ of\ adulthood)} * 100\%$$

To collect data on maximum lifespans (MAL) and age of sexual maturity/adulthood (AoSM) on a given species or population, we first prioritized using the data on lifespan reported directly in the study. If the study did not report MAL or AoSM, we collected this data from multiple sources. These included large publicly available databases such as Animal Diversity Web (https://animaldiversity.org/), AnAge[6], and Pantheria[7] as well as from data in published articles that contained supplementary datasets on MAL and AoSM estimates of species included in our study. There were very few databases that contained MAL and AoSM for animals such as insects. As a result, peer reviewed, published articles in scientific journals were used for data on MAL and AoSM for such species. When values were reported as a survival curve, the maximum age of the curve where individuals were alive was used. When data was not available from any of these sources, we also contacted the corresponding authors of the papers in our meta-analysis for maximum lifespan estimates on the species. We prioritized collecting data for MAL and AoSM on males. When male-specific data was not available, species-specific data was collected (this was the case for many vertebrates whose data were obtained from large datasets/databases). We never used female-specific data for any species. If a species had data on MAL and AoSM from multiple sources, these estimates were averaged, and the average was then used as the maximum lifespan of the species. This averaging was done so that exceptionally large values that might be unusual for a species (e.g. red junglefowl maximum lifespan is reported as 30 years on AnAge, which is quite unusual), did not bias our dataset. Additionally, when data from multiple sources was available, we ensured that we used data from males which most closely matched the rearing conditions and morphs of the species/study males in our meta-analysis. For insects, age of adulthood was considered as the age of eclosion/ last moult. Note that for species which had domesticated as well as wild-derived populations in our dataset (e.g. red junglefowl vs chicken, both of which were called *Gallus gallus* in our phylogeny), we collected their lifespan and sexual maturity data separately.

We tested the association between maximum and average lifespans collected for each species. This was high (R sq. = 0.85). Thus, maximum lifespan was used because it was available for a greater number of species than average lifespan, and less affected by juvenile mortality.

Supplementary notes 7: Moderators tested in our meta-regression models and their ranges/levels

Moderator	Levels
Proportion maximum adult lifespan sampled	0 to 100%
Ejaculate collection method	Male has control: males mated to female and female dissected/weighed post-insemination, male masturbated via dummy female, natural spawning<br>Male does not have control: Catheter, Males dissected, electroejaculation, males massaged with abdominal pressure
Taxonomic Class	Reptilia, Prosomapoda, Monogononta, Mammalia, Malacostraca,<br>Insecta, Hexanauplia, Gastropoda, Clitellata, Chromadorea, Aves, Arachnida, Amphibia, Actinopterygii
Population (i.e. Setting)	Laboratory, Domestic, Captive, Wild
Method of age estimation	Direct/ Indirect (i.e. inferred from body condition)
Trait	Concentration, DNA damage, Ejaculate size, Corrected quantity, Mitochondria, Morphology, Motility, Number of sperm, Oxidative stress, Length, Velocity, Viability
Longitudinal sampling	Yes/Semi (when only a subset of males were measured repeatedly)/No
Experimental	Yes: manipulated something in addition to male age, or assigned males to specific age groups at the start of the experiment to a target age class<br>No: observational sampling of the available age class distributions opportunistically
Gonadosomatic index	0 to 100 %
“Unnatural” manipulations	Yes/No
Cold storage of ejaculates (i.e. stored at <5°C)	Yes /No (only for sperm motility, viability, and velocity)

Supplementary notes 8: Definitions of population type

Captive: Managed study population which lives in an enclosed environment, and used in a captive breeding program, captive breeding centre, wildlife conservation research centre, or zoo. The webpage of the reported institutes where the study was conducted was checked when unsure, to ascertain whether it fell into this category.

Lab: Study population maintained under or adapted to lab conditions (for at least their adult life), living in an enclosed environment (even if collected in the wild pre-adulthood), but not used for a captive breeding program.

Domestic: Study population descended from a line of deliberately artificially selected individuals (inferred), or raised on commercial farms whose purpose is using animals for direct human consumption (e.g. as food, clothing, protection).

Wild: Was born in the wild/lives in natural unenclosed environments and was caught from the wild (post-adulthood) for the study.

Supplementary notes 9: "Unnatural" manipulations

"Unnatural" manipulations were defined as conditions experienced by males outside their physiological range (as defined in the study) and as conditions not typically experienced by healthy individuals (in the study populations). These manipulations also had a well-defined control in the study. These are as follows: pharmacological interventions such as toxins, chemicals, or medicines (Control: no pharmacological intervention); temperature manipulations (Control: standard temperature as defined in the study); radiation (Control: no radiation); genetic mutations/mutant lines with specific knocked out genes (Control: Wild type, or control lines as defined in the study); 24 hour dark or 24 hour light circadian durations (Control: 12:12 hour light durations); infection/disease (Control: no infection or disease); inbreeding (Control: Outbreeding); dietary or protein restriction (Control: Standard diet as defined in the study).

Other types of manipulations, such as sperm storage durations, mating history of males, seasons, male social status, or female age were not considered to be outside the typical range of conditions experienced by males, nor did they have an easy to define control, thus were not defined as "unnatural".

Supplementary notes 10: Calculation of effect sizes

We calculated Fisher's z transformed correlation coefficient (Zr) values from correlation coefficients (r) using the formula[4]: Zr = 0.5*(log(1+ r) - log(1-r)). Because r values were rarely ever directly reported in studies, we had to calculate these indirectly. For studies with two age groups, we calculated correlation coefficients (r) using standardized mean differences (SMD). When there were more than two age groups, we calculated correlation coefficients using a simulation. For studies where only test statistics were reported, we calculated correlation coefficients using test-specific formulae

A. Two-age groups

To calculate correlation coefficients from studies which reported comparisons between two age groups, we first calculated a standardized mean difference (SMD). SMD here, was calculated using the package *metafor* in R[8] with the function *escalc*. SMD provides the true strength of an effect by dividing the difference in means between two groups, by their pooled standard deviation. We used the following formula:

SMD = $\frac{(Mean\ (old) - Mean\ (young))}{S\ pooled}$

$$S_{pooled} = \sqrt{\frac{(n_{old} - 1)S_{old}^2 + (n_{young} - 1)S_{young}^2}{n_{old} + n_{young} - 2}}$$

where Mean (o) and (y) and means of the old and young age groups respectively, and S pooled is the pooled standard deviation. These SMD values were then converted to a correlation coefficient using the function *convert_d2r* in the package *meta*[9].

B. Multiple (>2) age groups

To calculate effect sizes from studies which reported means and standard deviations from more than two age groups, we used a simulation (with 1000 iterations). This simulation resulted in a correlation coefficient between the age of males and their means at each age, while weighting the means by their standard deviations. To test for consistency between effect size outcomes from the simulation and the outcomes from SMD, we additionally calculated correlation coefficient for outcomes with only two-groups using the simulation. There was a very strong agreement between r values obtained from these two methods (i.e. SMD and simulation) (R sq.> 0.95).

C. Test statistics[10,11]

1. For converting "t" from independent t-test into *r*

$$r = \frac{t}{\sqrt{(t^2 + degrees\ of\ freedom)}}$$

2. For converting F from ANOVA or ANCOVA with 1 degree of freedom to *r*

$$r = \sqrt{(\frac{F}{F + N - 2})}$$

Where N is the sample size of unique number of males

3. For converting spearman's rho to *r*

$$r = 2 * \sin((\pi * \rho) / 6)$$

4. From Mann Whitney-U to *r*

$$r = \frac{1 - (2 * U)}{n1 * n2}$$

   Where n1 and n2 are sample sizes of the younger and older age groups respectively

5. From z-score to *r*

$$r = \frac{z}{\sqrt{N}}$$

   Where N is the sample size of unique number of males

6. For Converting T from Seigel's T test, and converting P values from Mann Whitney U test, to *r*, the Campbell Collaboration website was used (https://www.campbellcollaboration.org/research-resources/effect-size-calculator.html)
7. For converting R squared and adjusted R squared values to *r*

$$r = \sqrt{R}^2$$

8. For converting Chisq. values from Chisq. test with one degree of freedom, to *r*

$$r = \sqrt{(\frac{Chi\ sq.}{N})}$$

9. For converting Pearson's or Spearman's correlation coefficient (r) to *r*

$$r = \mathrm{r}$$

<u>Multiplier and signs</u>

If an increase in a trait signified a deleterious effect with increasing age, for example, an increase in sperm abnormal morphology, or sperm DNA damage, we assigned it a multiplier of "-1", whereas if increase in a trait suggested improvement with age, we assigned it a positive multiplier, i.e. "+1". Similarly, when a test statistic was reported (e.g. R sq., correlation coefficient. F values from ANOVA), where older males had worse sperm or ejaculates than younger males, we assigned it a negative multiplier of "-1". Conversely, if older males had better sperm or ejaculates than younger males, we assigned it a multiplier of "+1". Thus, for all the effect sizes in our models, a negative sign indicated reproductive senescence with increasing age, while a positive sign indicated reproductive improvement with increasing age.

<u>Comparing calculation methods</u>

To ensure that the three different effect size calculation methods did not affect the overall outcome in our meta-analysis, we compared the meta-analytical mean obtained from each method (*i.e.* SMD, simulation, and test-statistics). These did not differ from each other (see Supplementary Fig. 24), hence we analysed effect sizes calculated from SMD, simulation, and test-statistics together in subsequent models.

Supplementary notes 11: Quadratic effects of age

Our meta-analyses used an effect size (Zr), which assumes a linear relationship between independent (age) and dependent (ejaculate traits) variables[12]. Thus, to test whether the effects of advancing male age on ejaculate traits are curvilinear in shape, we created linear mixed models (LMM) in the package *lme4*[13] and *lmerTest*[14], because meta-regression models, to our knowledge, cannot model non-linear effect sizes.

Our LMM was conducted on three traits (percent motile, percent morphologically normal, and percent viable sperm), which were measured on the same scale across studies and species. These three traits were thus already "standardized" thus could be compared across studies the way an effect size is. We standardized each of the different ages at which males were sampled at, as the proportion of maximum adult lifespan of the species (independent variable). We standardized the traits (response variable) by calculating the proportion of: morphologically normal sperm (85 studies), viable sperm (137 studies), and motile sperm (81 studies), and analyzed them in three separate models.

We included linear and quadratic effects of [standardized] age (covariates), with population type (i.e, lab, captive, domestic, wild) and taxonomic class as fixed effects. We also used the sample size of males within each age class (log transformed) as weights in the LMM. We included effect size ID, cohort ID, and study ID as random effects. Our models met assumptions of homoscedasticity and normality of residuals, checked using the *stats* package[15].

We found significant quadratic effects of [standardised] male age on [standardised] ejaculate traits (Supplementary Fig. 7). Specifically, we found significant quadratic effects for age-dependent changes in: percent morphologically normal sperm ($t = -3.023$, $P = 0.003$, $DF = 346$); percent motile sperm ($t = 2.296$, $P = 0.022$, $DF = 589$); and percent viable sperm ($t = -3.909$, $P < 0.001$, $DF = 437$).

Supplementary notes 12: Sensitivity analysis and publication bias

<u>Sensitivity analysis</u>

To test for robustness of our full and null models, we created a variance-covariance matrix (VCV) of correlation values between effect size ID and cohort ID that replaced the variance argument[16] in both, null and full models. The results obtained for the null model or full model were not different between the model that used the VCV matrix versus the one that did not, thus in the main text, we present the model without the VCV matrix throughout (See "metaA HTML" file for model outputs).

<u>Publication bias</u>

We tested for publication bias using various methods[16,17]. We first visually evaluated symmetry in a funnel plot with effect sizes (Zr) on the X axis, and the inverse of the standard error (1/SE) of the effect size (i.e. precision) on the Y axis (note that for Zr, precision is proportional to sample sizes). Second, we conducted a multi-level meta-regression to evaluate whether the size of a study and its year of publication influences effect sizes (i.e. small study bias and time-lag bias) by including both, standard error of effect sizes and year of publication as moderators, and effect size ID, cohort ID, study ID, species name, and phylogeny as random effects. Third, we created a funnel plot with the average of effect sizes from each study (X axis) against precision (Y axis), and tested for funnel asymmetry using a trim-and-fill method[16]. Finally, we created a selection model to test whether the probability of selecting a study depended on the significance of its effect size[18].

We found little evidence for publication bias. Visual inspection of funnel plots did not indicate any asymmetrical distribution of effect sizes around zero, indicating no evidence for publication bias (Supplementary Fig. 17 below, test for funnel plot asymmetry: P = 0.992). Our multi-level meta-regression publication bias test indicated no evidence for a small study bias (t = 7.21, P = 0.721, DF = 1810; Supplementary Fig. 18) but significant evidence for a time-lag bias, with more recent studies being more likely to show reproductive senescence (t = -2.34, P = 0.019, DF = 1810, Supplementary Fig. 18). We found no evidence for missing studies (missing studies on right= 0, SE = 10.94) in our trim-and-fill model that used one averaged effect size from each study (n = k = 379). Finally, we did not find a bias toward more significant effect sizes in our selection model (Supplementary Fig. 19).

References:

1. Rolff, J., Johnston, P.R., Reynolds, S., 2019. Complete metamorphosis of insects. Phil. Trans. R. Soc. B 374, 20190063. https://doi.org/10.1098/rstb.2019.0063
2. Rohtagi, A., 2014. WebPlotDigitizer user manual version 3.4. URL http://arohatgi. info/WebPlotDigitizer/app.
3. Pick, J.L., Nakagawa, S., Noble, D.W.A., 2019. Reproducible, flexible and high-throughput data extraction from primary literature: The METADIGITISE R package. Methods Ecol Evol 10, 426–431. https://doi.org/10.1111/2041-210X.13118
4. Nakagawa, S., Cuthill, I.C., 2007. Effect size, confidence interval and statistical significance: a practical guide for biologists. Biological Reviews 82, 591–605. https://doi.org/10.1111/j.1469-185X.2007.00027.x
5. Wan, X., Wang, W., Liu, J., Tong, T., 2014. Estimating the sample mean and standard deviation from the sample size, median, range and/or interquartile range. BMC Med Res Methodol 14, 135. https://doi.org/10.1186/1471-2288-14-135
6. De Magalhães, J.P., Costa, J., 2009. A database of vertebrate longevity records and their relation to other life-history traits. Journal of Evolutionary Biology 22, 1770–1774. https://doi.org/10.1111/j.1420-9101.2009.01783.x
7. Jones, K.E., Bielby, J., Cardillo, M., Fritz, S.A., O'Dell, J., Orme, C.D.L., Safi, K., Sechrest, W., Boakes, E.H., Carbone, C., Connolly, C., Cutts, M.J., Foster, J.K., Grenyer, R., Habib, M., Plaster, C.A., Price, S.A., Rigby, E.A., Rist, J., Teacher, A., Bininda-Emonds, O.R.P., Gittleman, J.L., Mace, G.M., Purvis, A., 2009. PanTHERIA: a species-level database of life history, ecology, and geography of extant and recently extinct mammals: Ecological Archives E090-184. Ecology 90, 2648–2648. https://doi.org/10.1890/08-1494.1
8. Viechtbauer, W., 2010. Conducting Meta-Analyses in *R* with the metafor Package. J. Stat. Soft. 36. https://doi.org/10.18637/jss.v036.i03
9. Schwarzer, G. and Schwarzer, M.G., 2012. Package 'meta'. *The R foundation for statistical computing*, *9*, p.27.
10. Koricheva, J., Gurevitch, J., Mengersen, K. (Eds.), 2013. Handbook of meta-analysis in ecology and evolution. Princeton University Press, Princeton.
11. Polanin, J.R., Snilstveit, B., 2016. Converting between effect sizes. Campbell Systematic Reviews 12, 1–13. https://doi.org/10.4073/cmpn.2016.3
12. Baker, W.L., Michael White, C., Cappelleri, J.C., Kluger, J., Coleman, C.I., From the Health Outcomes, Policy, and Economics (HOPE) Collaborative Group, 2009. Understanding heterogeneity in meta-analysis: the role of meta-regression. International Journal of Clinical Practice 63, 1426–1434. https://doi.org/10.1111/j.1742-1241.2009.02168.x
13. Bates, D., Mächler, M., Bolker, B., Walker, S., 2014. Fitting Linear Mixed-Effects Models using lme4. https://doi.org/10.48550/ARXIV.1406.5823
14. Kuznetsova, A., Brockhoff, P.B., Christensen, R.H.B., 2017. lmerTest Package: Tests in Linear Mixed Effects Models. J. Stat. Soft. 82. https://doi.org/10.18637/jss.v082.i13
15. R Development Core Team. (2022) R: a language and environment for statistical computing. R Foundation for Statistical Computing, Vienna.
16. Nakagawa, S., Lagisz, M., Jennions, M.D., Koricheva, J., Noble, D.W.A., Parker, T.H., Sánchez-Tójar, A., Yang, Y., O'Dea, R.E., 2022. Methods for testing publication bias in ecological and evolutionary meta-analyses. Methods Ecol Evol 13, 4–21. https://doi.org/10.1111/2041-210X.13724
17. Nakagawa, S., Santos, E.S.A., 2012. Methodological issues and advances in biological meta-analysis. Evol Ecol 26, 1253–1274. https://doi.org/10.1007/s10682-012-9555-5
18. Marks-Anglin, A., Chen, Y., 2020. A historical review of publication bias. Res Syn Meth 11, 725–742. https://doi.org/10.1002/jrsm.1452
