# Supplementary material for: Meta-analysis shows no consistent evidence for senescence in ejaculate traits across animals
Source: Nat Commun. 2024 Jan 16;15:558. doi: 10.1038/s41467-024-44768-4 (PMC10791739; doi:10.1038/s41467-024-44768-4)
Supplement: Supplementary file 3 — Reporting Summary [file 41467_2024_44768_MOESM3_ESM.pdf]

nature portfolio

Corresponding author(s): Krish Sanghvi, Regina Vega-Trejo, Tommaso Pizzari, Irem Sepil

Last updated by author(s): Dec 13, 2023

Reporting Summary

Nature Portfolio wishes to improve the reproducibility of the work that we publish. This form provides structure for consistency and transparency in reporting. For further information on Nature Portfolio policies, see our Editorial Policies and the Editorial Policy Checklist.

Statistics

For all statistical analyses, confirm that the following items are present in the figure legend, table legend, main text, or Methods section.

n/a	Confirmed	
☐	☒	The exact sample size (n) for each experimental group/condition, given as a discrete number and unit of measurement
☐	☒	A statement on whether measurements were taken from distinct samples or whether the same sample was measured repeatedly
☐	☒	The statistical test(s) used AND whether they are one- or two-sided<br>*Only common tests should be described solely by name; describe more complex techniques in the Methods section.*
☐	☒	A description of all covariates tested
☐	☒	A description of any assumptions or corrections, such as tests of normality and adjustment for multiple comparisons
☐	☒	A full description of the statistical parameters including central tendency (e.g. means) or other basic estimates (e.g. regression coefficient) AND variation (e.g. standard deviation) or associated estimates of uncertainty (e.g. confidence intervals)
☐	☒	For null hypothesis testing, the test statistic (e.g. F, t, r) with confidence intervals, effect sizes, degrees of freedom and P value noted<br>*Give P values as exact values whenever suitable.*
☒	☐	For Bayesian analysis, information on the choice of priors and Markov chain Monte Carlo settings
☒	☐	For hierarchical and complex designs, identification of the appropriate level for tests and full reporting of outcomes
☐	☒	Estimates of effect sizes (e.g. Cohen's d, Pearson's r), indicating how they were calculated

Our web collection on statistics for biologists contains articles on many of the points above.

Software and code

Policy information about availability of computer code

Data collection	Study abstracts were screened on Rayyan and abstrackr. WebPlotDigitizer was used to obtain data from figures. Data on phylogenetic relatedness were extracted from R open tree of life. All data collected from each study, including supplementary data, can be accessed from the OSF link: https://osf.io/dk8sq, and is also provided in the source data file
Data analysis	R and Rstudio, were used for all data analysis. All code needed to run the analyses can be accessed from the OSF link: https://osf.io/dk8sq, with DOI: 10.17605/OSF.IO/DK8SQ. These are under the file names Meta.Ageing.AnalysesCode.html and Meta.Ageing.AnalysesCode.Rmd, on OSF.

For manuscripts utilizing custom algorithms or software that are central to the research but not yet described in published literature, software must be made available to editors and reviewers. We strongly encourage code deposition in a community repository (e.g. GitHub). See the Nature Portfolio guidelines for submitting code & software for further information.

Data

Policy information about availability of data

All manuscripts must include a data availability statement. This statement should provide the following information, where applicable:

- Accession codes, unique identifiers, or web links for publicly available datasets
- A description of any restrictions on data availability
- For clinical datasets or third party data, please ensure that the statement adheres to our policy

All associated data can be found at OSF https://osf.io/dk8sq with the identifier 10.17605/OSF.IO/DK8SQ. Source data and supplementary material are provided with

this paper. The list of all 379 studies used in the meta-analysis can be found under the file name “Studies included in meta-analysis.pdf” on OSF. Raw data is under the file name “raw_data.csv” on OSF.

Research involving human participants, their data, or biological material

Policy information about studies with human participants or human data. See also policy information about sex, gender (identity/presentation), and sexual orientation and race, ethnicity and racism.

Reporting on sex and gender	NA
Reporting on race, ethnicity, or other socially relevant groupings	NA
Population characteristics	NA
Recruitment	NA
Ethics oversight	NA

Note that full information on the approval of the study protocol must also be provided in the manuscript.

Field-specific reporting

Please select the one below that is the best fit for your research. If you are not sure, read the appropriate sections before making your selection.

☐ Life sciences ☐ Behavioural & social sciences ☒ Ecological, evolutionary & environmental sciences

For a reference copy of the document with all sections, see nature.com/documents/nr-reporting-summary-flat.pdf

Ecological, evolutionary & environmental sciences study design

All studies must disclose on these points even when the disclosure is negative.

Study description	We conducted a meta-analysis on 379 studies (number of experimental units), to quantify the effects of advancing male age on ejaculate traits across 157 species. We had a total of 1814 effect sizes from these studies. Our moderators (akin to treatments) were: taxonomic class, ejaculate trait, proportion of maximum adult lifespan sampled, whether or not males had control over ejaculation, population type, sampling method of males, method of age estimation, whether or not a study was experimental, and whether or not males underwent “unnatural” manipulations, and male gonadosomatic index.
Research sample	Our sample sizes included 1814 effect sizes, 379 studies, 624 cohorts, and 157 species. Our sample sizes were not pre-determined, and were instead determined by the number of relevant studies we were able to obtain to be included in our meta-analysis, the number of effect sizes that could be obtained from each study, and the number of cohorts and species that the studies represented. The references for each of the studies included in the meta-analysis can be found at OSF https://osf.io/dk8sq with the identifier 10.17605/OSF.IO/DK8SQ, under the file name “Studies included in meta-analysis.pdf”. The raw dataset of all studies, can be found at OSF https://osf.io/dk8sq with the identifier 10.17605/OSF.IO/DK8SQ, under the file name “raw_data.csv”. The sample in our meta-analysis represents non-human animals of different ages, for whom data on ejaculate traits were available through our search terms and inclusion criteria.
Sampling strategy	Our sample sizes were determined by the number of studies we were able to obtain to be included in our meta-analysis, the number of effect sizes that could be obtained from each study, and the number of species present in these studies. Sample sizes were not pre-determined. Within each study, sample sizes for each age class was the number of males measured by that study, for a given trait and cohort, in a specific age class. These data can be found at OSF https://osf.io/dk8sq with the identifier 10.17605/OSF.IO/DK8SQ, under the file name “raw_data.csv”.
Data collection	KS recorded whether to include studies or not using a pre-defined criterion, on an excel sheet, which can be found at OSF https://osf.io/dk8sq with the identifier 10.17605/OSF.IO/DK8SQ, under the file name “screening_allStudies.xlsx”. SJLG screened 50% of these studies to ensure high repeatability of abstract screening and full-text screening procedures (91% agreement, Cohen's Kappa = 0.56). KS recorded the data used for the meta-analytical models from all 379 studies on an excel file, found at OSF https://osf.io/dk8sq/?view_only=) with the identifier 10.17605/OSF.IO/DK8SQ, under the file name “raw_data.csv”. RVT checked 20 studies to ensure high repeatability of the data extraction process (98% agreement, Cohen's Kappa = 0.96).
Timing and spatial scale	The search on SCOPUS was done on 21st January, 2021 (year range of studies: 1923 to 2021), and on web of science on 27th March, 2021 (year range of studies: 1951 to 2021), accessed from the university of oxford server. Studies obtained from these search engines were screened between April 2021 and February 2022, and data was extracted between February 2022 and November 2022. Because ours was not an experimental study, the periodicity of data extraction from collected studies would not affect the results, thus is irrelevant to our meta-analysis.
Data exclusions	For a study to be included in our analysis, some selection criteria had to be fulfilled during the abstract and full-text screening stages. When screening abstracts, the study had to be a research article (not a review, meta-analysis, or case study), written in English, on non-human animals, and quantify ejaculate traits in males of different ages. When screening full-texts, the study needed to contain: data on the effects of male age on ejaculate traits, non-overlapping age groups of males, and appropriate data for calculation of

	effect sizes. We only included studies where at least two age groups of adult males could be compared (see Supplementary section 3 for our definition of "adults"). All other studies were excluded. This inclusion criterion allowed us to obtain only relevant papers to our meta-analysis aims. Whether a study was included or not, and the reasons for exclusion, can be found at at OSF https://osf.io/dk8sq with the identifier 10.17605/OSF.IO/DK8SQ, under the file name "screening_AllStudies.xlsx".
Reproducibility	Our study was not experimental, thus did not have replications. However, SJLG screened 50% of study abstracts to ensure high repeatability of abstract screening and full-text screening procedures (91% agreement, Cohen's Kappa = 0.56); and RVT checked 20 included studies to ensure high repeatability of the data extraction process (98% agreement, Cohen's Kappa = 0.96).
Randomization	Randomization was not relevant to our study as our study was not experimental, and was a meta-analysis, where data is extracted from already conducted experimental studies. However, to test for biases in our included dataset, we comducted various publication bias tests and sensitivity analyses (described in the paper). To control for covariances between moderators, in our full model, we included all moderators present in >75% effect sizes, as this would test for the effects of each moderator after accounting for the effects of all other co-varying moderators.
Blinding	During the screening process, two authors were involved, blind to each other's decision. The decisions of the authors were compared in order to determine the selection process was uniform between authors.

Did the study involve field work? ☐ Yes ☒ No

Reporting for specific materials, systems and methods

We require information from authors about some types of materials, experimental systems and methods used in many studies. Here, indicate whether each material, system or method listed is relevant to your study. If you are not sure if a list item applies to your research, read the appropriate section before selecting a response.

Materials & experimental systems

n/a	Involved in the study
☒	☐ Antibodies
☒	☐ Eukaryotic cell lines
☒	☐ Palaeontology and archaeology
☒	☐ Animals and other organisms
☒	☐ Clinical data
☒	☐ Dual use research of concern
☒	☐ Plants

Methods

n/a	Involved in the study
☒	☐ ChIP-seq
☒	☐ Flow cytometry
☒	☐ MRI-based neuroimaging
